# A cluster of mesopontine GABAergic neurons suppresses REM sleep and curbs cataplexy

**DOI:** 10.1038/s41421-022-00456-5

**Published:** 2022-10-25

**Authors:** Ze-Ka Chen, Hui Dong, Cheng-Wei Liu, Wen-Ying Liu, Ya-Nan Zhao, Wei Xu, Xiao Sun, Yan-Yu Xiong, Yuan-Yuan Liu, Xiang-Shan Yuan, Bing Wang, Michael Lazarus, Yoan Chérasse, Ya-Dong Li, Fang Han, Wei-Min Qu, Feng-Fei Ding, Zhi-Li Huang

**Affiliations:** 1 Department of Pharmacology, School of Basic Medical Sciences; State Key Laboratory of Medical Neurobiology and MOE Frontiers Center for Brain Science, Institutes of Brain Science, Fudan University, Shanghai, China; 2 Department of Anatomy, Histology and Embryology, School of Basic Medical Sciences, Fudan University, Shanghai, China; 3 ENT Institute and Otorhinolaryngology Department, Affiliated Eye and ENT Hospital, State Key Laboratory of Medical Neurobiology, Fudan University, Shanghai, China; 4 International Institute for Integrative Sleep Medicine (WPI-IIIS), University of Tsukuba, Tsukuba, Ibaraki Japan; 5 Sleep Medicine Center, Department of Respiratory and Critical Care Medicine, Peking University People’s Hospital, Beijing, China

**Keywords:** Circadian rhythms, Mechanisms of disease

## Abstract

Physiological rapid eye movement (REM) sleep termination is vital for initiating non-REM (NREM) sleep or arousal, whereas the suppression of excessive REM sleep is promising in treating narcolepsy. However, the neuronal mechanisms controlling REM sleep termination and keeping sleep continuation remain largely unknown. Here, we reveal a key brainstem region of GABAergic neurons in the control of both physiological REM sleep and cataplexy. Using fiber photometry and optic tetrode recording, we characterized the dorsal part of the deep mesencephalic nucleus (dDpMe) GABAergic neurons as REM relatively inactive and two different firing patterns under spontaneous sleep–wake cycles. Next, we investigated the roles of dDpMe GABAergic neuronal circuits in brain state regulation using optogenetics, RNA interference technology, and celltype-specific lesion. Physiologically, dDpMe GABAergic neurons causally suppressed REM sleep and promoted NREM sleep through the sublaterodorsal nucleus and lateral hypothalamus. In-depth studies of neural circuits revealed that sublaterodorsal nucleus glutamatergic neurons were essential for REM sleep termination by dDpMe GABAergic neurons. In addition, dDpMe GABAergic neurons efficiently suppressed cataplexy in a rodent model. Our results demonstrated that dDpMe GABAergic neurons controlled REM sleep termination along with REM/NREM transitions and represented a novel potential target to treat narcolepsy.

## Introduction

The periodic rhythm and continuity are essential for sleep physiology^1,2^. For continuous sleep in healthy human, rapid eye movement (REM) sleep follows non-REM (NREM) sleep several times during a typical night of sleep, while reductions in NREM sleep, disinhibition of REM sleep, and insomnia usually accompanied by non-consolidated sleep^3^. REM sleep was first described in 1953 by Kleitman and Aserinsky as regularly occurring “active sleep” in human infants, which is distinct from the quiescent sleep periods known as NREM sleep^4^. Of the rapid-eye-movements produced by bursting of oculomotor muscles, REM sleep is also characterized by desynchronized cortical electroencephalogram (EEG), high-amplitude theta waves, and muscle atonia^5^. REM sleep is associated with brain development^6,7^ and memory^8,9^. Dysregulation of REM sleep leads to numerous sleep disorders, including narcolepsy^10,11^. Although previous studies have described several brain regions that contribute to the promotion of REM sleep, such as the sublaterodorsal nucleus (SLD), ventral medulla, lateral hypothalamus (LH), etc^12–15^, the core mechanisms controlling the termination of REM sleep and REM/NREM alternations have been paid less attention to. Previous studies reveal that the monoaminergic neurons in the brainstem cease firing specifically during REM sleep, including serotonergic neurons from the raphe nuclei and noradrenergic neurons from the locus coeruleus (LC)^16,17^. Since the 1970s, non-monoamine-based inhibitory neurons in the ventrolateral periaqueductal gray matter/the deep mesencephalic nucleus (vlPAG/DpMe) have been reported as key parts of REM sleep inhibition^18–20^. However, the vital mechanisms of REM-sleep cessation are still controversial, and the neural mechanism underlying the maintenance of the REM sleep necessary for living and termination of endless REM sleep or REM-like pathological sleep remains largely uncertain.

In 1975, Petitjean et al. reported that electrical destruction of the dorsal norepinephrine bundle (including vlPAG/DpMe) in the mesencephalon induced an increase in REM sleep^21^. Consistently, chemical lesion of the area ventrolateral part to the vlPAG, the dorsal part of the deep mesencephalic nucleus (dDpMe, also called lateral pontine tegmentum) increased excessive REM sleep in mice^20^. Boissard et al. found the vlPAG/DpMe GABAergic neurons projecting to the SLD, the key REM sleep center^22^. Furthermore, dDpMe GABAergic (dDpMe^GABA^) neurons were found to be activated by REM-sleep deprivation^23^. Recent research reported a cluster of neurons expressing Atoh1 derived from the hindbrain that inhibited REM sleep by the dDpMe^18^. In humans, lesions of pons containing the dDpMe caused cataplexy and visual hallucinations due to excessive REM sleep^24,25^. However, direct evidence proving dDpMe^GABA^ neurons as the essential elements in REM sleep regulation and REM-related disorders was still not sufficient. It was yet to be reported that how the real-time manipulation of dDpMe^GABA^ neurons would affect REM, NREM, and wake state transitions.

In the current study, we applied fiber photometry and multichannel recording in vivo to investigate the spontaneous activity of dDpMe^GABA^ neurons corresponding to REM sleep. In addition, we used optogenetics^26^, and RNA interference to reveal the crucial role of dDpMe^GABA^ neurons in REM-sleep termination and sleep continuity. Finally, we applied the optogenetic manipulation in a virus-based orexin neuron-lesioned rodent model to investigate the cure effect of dDpMe^GABA^ neurons on cataplexy. These findings may provide further insights into the pathophysiology of narcolepsy and reveal potential therapeutic avenues for sleep disorders.

## Results

### Spontaneous activity of dDpMe^GABA^ neurons across different brain state transitions

**Fig. 1:**
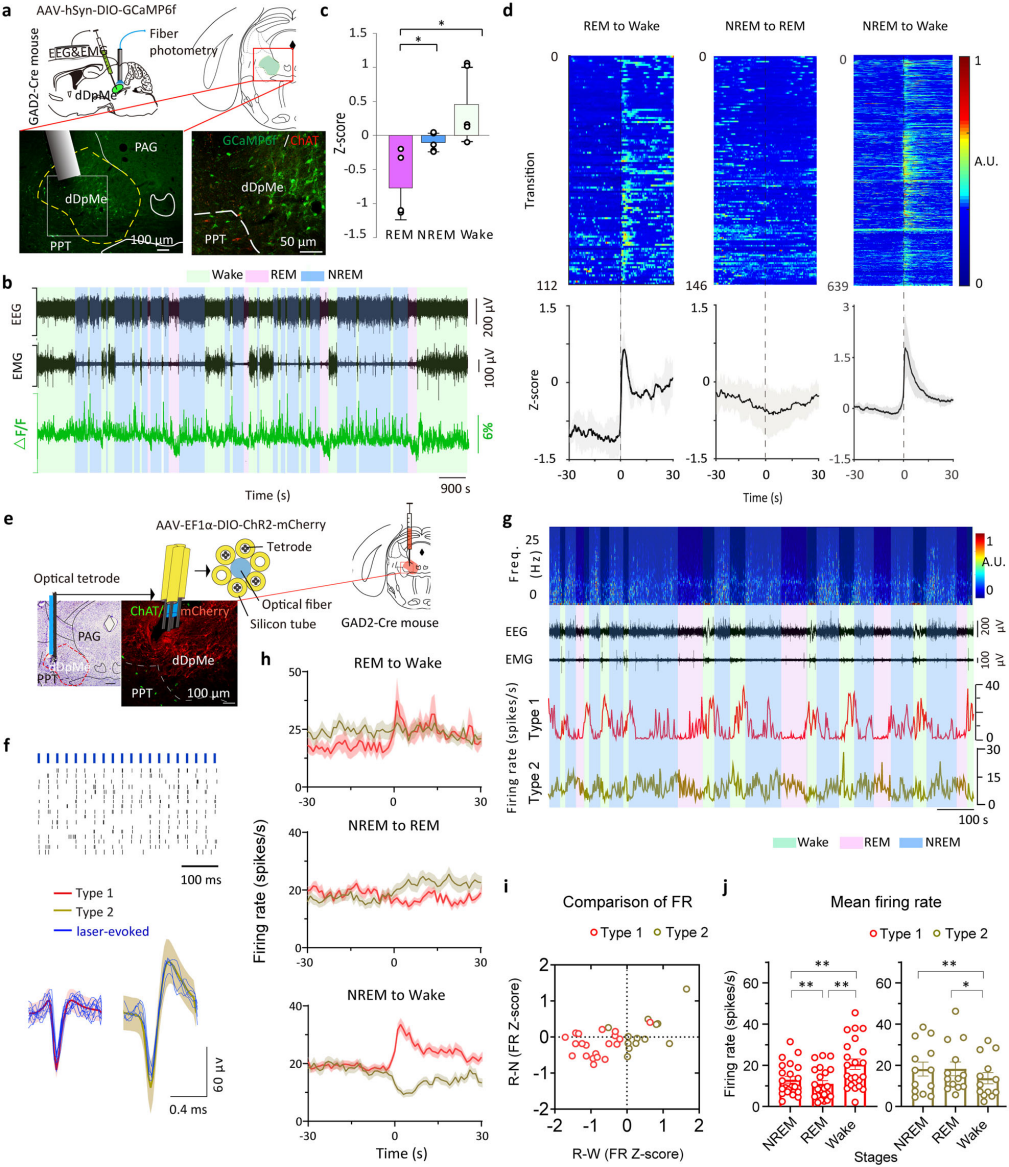
Activity of dDpMe^GABA^ neurons across different brain state transitions. **a** Schematic showing the fiber photometry used to assess GCaMP6f fluorescence of the dDpMe in GAD2-IRES-Cre mice with simultaneous polysomnographic recordings. Drawings of irregular-shaped superimposed viral expressing sites with circle-shaped micropipette tips (*n* = 5). **b** Raw traces of GCaMP6f fluorescence changes that were associated with different brain states. **c** Mean normalized fluorescence (*z*-score) during wake, NREM sleep, and REM sleep (*n* = 5 mice), taking the mean of 8 sessions per mouse; one-way ANOVA between brain states: *F* (1.324, 5.295) = 17.12, *P* = 0.0065; *P < 0.05, **P < 0.01, followed by Tukey’s multiple comparison test, *P*_(REM–Wake)_ = 0.022 and *P*_(REM–NREM)_ = 0.018. **d** Ca^2+^ normalized signals (*z*-score) associated with transitions among brain states. Top, individual transitions with color-coded fluorescence intensity; bottom, *z*-score of fluorescence changes from all transitions expressed as means (blue trace) ± SEM (shading). **e** Schematic showing the multichannel recording in vivo used to assess signal units by optic tetrodes with simultaneous polysomnographic recordings (*n* = 35). Typical fluorescent images representing the dDpMe labeled by ChR2-mCherry near the ChAT-labeled PPT (Green) and recording sites identified by electrolytic lesions stained by Nissl. **f** Example unit. Top, spike raster showing multiple trials of 470 nm laser stimulation. Bottom, raw trace showing spontaneous and laser-evoked spikes. Red, mean spontaneous firing rate of Type 1 neurons; yellow-green, mean spontaneous firing rate of Type 2 neurons; blue, laser-evoked spikes. **g** The firing rate of the neuron with the EEG spectrogram, EMG trace, and brain states (color coded). Freq, Frequency. There are two types of neurons among all identified GABAergic units in the dDpMe. Red, Type 1; yellow-green, Type 2. **h** The variation of identified dDpMe GABAergic neuronal firing rates at state transitions. To display the whole process of each phase, we picked up 30 s before and after each time point of state transitions, and then the firing rates averaged during REM sleep, wake, or NREM sleep were within each segments. Shading indicates ± SEM. **i** REM-NREM (R-N) vs REM-wakefulness (R-W) activity difference. **j** Mean firing rate of two-type dDpMe GABAergic neurons among brain states. Assessed by one-way ANOVA followed by Tukey’s multiple comparison test, *P < 0.05, **P < 0.01. Type 1: *n* = 22, *P*_(REM–Wake)_ = 2.0 × 10^−4^, *P*_(REM–NREM)_ = 9.2 × 10^−3^, and *P*_(Wake–NREM)_ = 2.6 × 10^−3^; Type 2: *n* = 13, *P*_(REM–Wake)_ = 0.028 and *P*_(Wake–NREM)_ = 2.6 × 10^−3^. dDpMe dorsal part of the deep mesencephalic nucleus, PAG periaqueductal gray, PPT pedunculopontine tegmental nucleus.

First, we assessed the population activity of dDpMe^GABA^ neurons across spontaneous sleep–wake states using fiber photometry technology^27,28^. To this end, we injected a Cre-dependent adeno-associated virus (AAV) encoding the fluorescent calcium indicator GCaMP6f into the dDpMe of GAD2-IRES-Cre knock-in mice (*n* = 5) (Fig. 1a and Supplementary Fig. S1a). The dDpMe is located in the mesopontine region of the brainstem as previous studies claimed, which is also called lateral pontine tegmentum^20,23,29^. Our results showed that GCaMP6f fluorescence signals were the lowest during REM sleep and higher during wakefulness or NREM sleep (Fig. 1b). The mean normalized fluorescence signals indicated that the activity of dDpMe^GABA^ neurons in REM sleep was lower than that in NREM sleep or wakefulness (Fig. 1c). When comparing the mean normalized fluorescence signal transitions across brain states, we found obvious alterations in the population activity of dDpMe GABAergic neurons (Fig. 1d and Supplementary Fig. S1b). Notably, the activity of these neurons increased swiftly across REM-to-wake and NREM-to-wake transitions, and decreased slightly across NREM-to-REM sleep transitions.

We next applied in vivo multichannel recording to identify whether dDpMe^GABA^ neurons have heterogeneity of neural activity. We recorded ChR2-tagged single-cell activity in GAD2-Cre mice during the sleep–wake cycle using optic tetrodes and electroencephalogram(EEG)/electromyography (EMG) signals synchronously (Fig. 1e and Supplementary Fig. S1c). Single units exhibiting reliable laser-evoked spiking at short latencies (<8 ms) were identified as GABAergic neurons. Typical traces of EEG/EMG and the firing rate showed that there were two types of ChR2-tagged neurons in the dDpMe (Fig. 1g) as Type 1 with short half-wavelength, low amplitude, and REM relatively inactive and Type 2 with long half-wavelength and high amplitude (Fig. 1f). Mean firing rate of Type 1 neurons showed a marked increase from REM→Wake, REM→NREM, NREM→Wake and a decrease from NREM→REM, Wake→NREM, whereas the activity of Type 2 neurons has the opposite change (Fig. 1h; Supplementary Fig. S1d). To quantify the relative firing rates of each neuron in different brain states, we calculated REM–Wake vs REM–NREM activity difference. Of the 35 identified GABAergic neurons, 22 neurons almost fell in the bottom left quadrant (Fig. 1i), indicating that these Type 1 neurons were mostly REM sleep relatively inactive. The other neurons were Type 2 neurons, which almost fell in the right quadrant. The mean firing rates of these neurons were more active during REM and NREM sleep than wakefulness (Fig. 1i). In addition, we stated that the firing patterns of Type 1 and Type 2 neurons were different as the firing rate of Type 1 neurons was the lowest in REM sleep whereas that of Type 2 neurons was the lowest in wakefulness versus NREM or REM sleep (Fig. 1j).

These findings reflect the physiological activity of GABAergic neurons in the dDpMe (including Type 1 and 2 neurons) changes upon transitions from REM sleep to NREM sleep or wakefulness. It suggests their participation in the regulation of REM sleep and state transitions.

### Activation of dDpMe^GABA^ neurons suppressed REM sleep and transferred REM sleep to NREM sleep

**Fig. 2:**
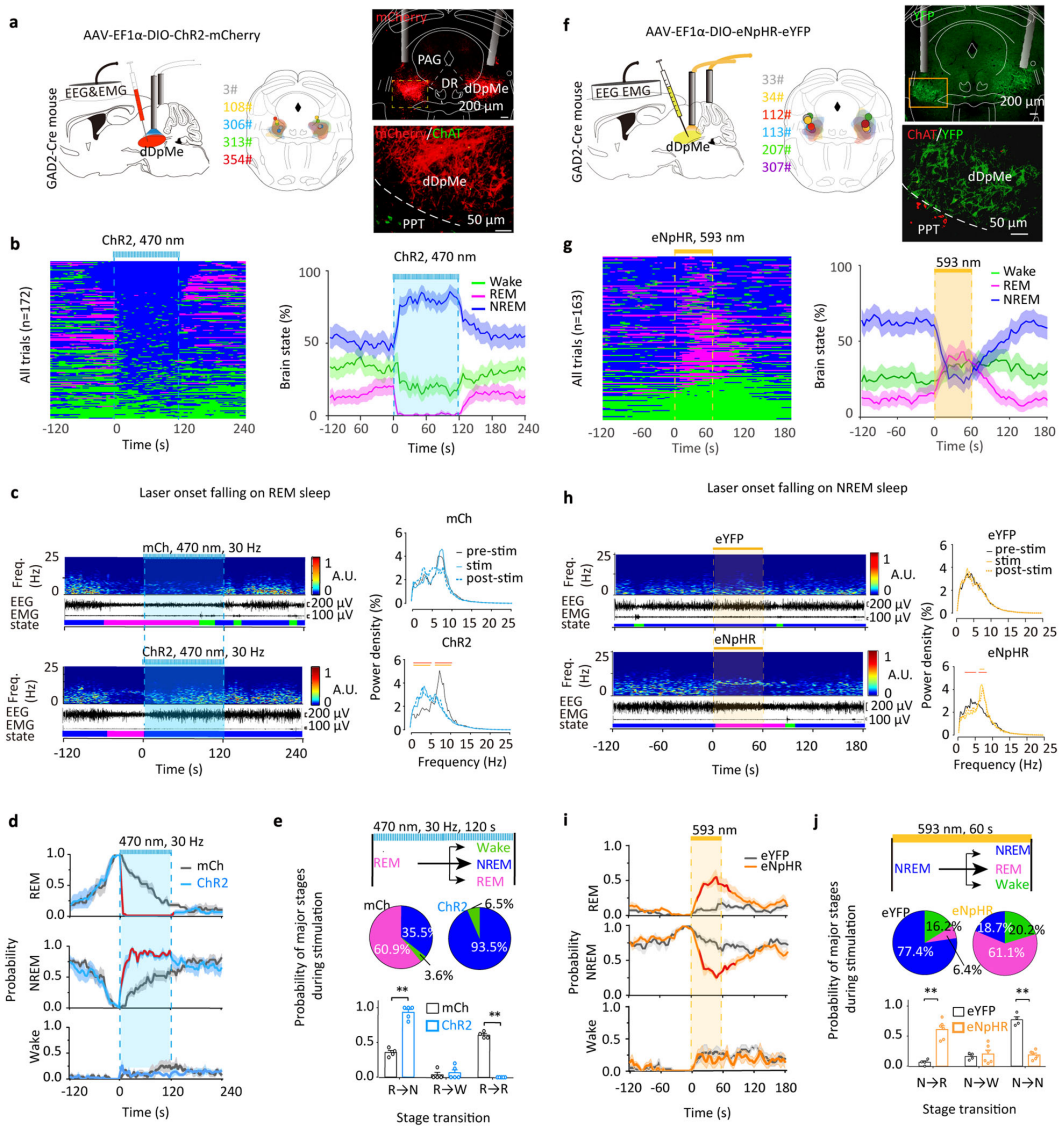
dDpMe^GABA^ neurons are essential for REM sleep suppression and REM-to-NREM transitions. **a** Schematic of optogenetic experiments with EEG/EMG recordings in a GAD2-IRES-Cre mouse with an AAV carrying Cre-dependent ChR2-mCherry injected into the dDpMe. Drawings of superimposed ChR2-mCherry-expressing sites with micropipette tips (*n* = 5, circles indicate tips and irregular shapes indicate expressing sites) and a typical fluorescent image representing the dDpMe labeled by ChR2-mCherry, dorsomedial to the ChAT-labeled PPT. **b** All trials (*n* = 172) and the probabilities of different brain states from five mice before, during, and after blue-light delivery (30 Hz, 120 s). **c** An example recording shows the EEG power spectrograms, EEG/EMG traces, and hypnograms after bilateral laser onset above the dDpMe in terms of REM sleep. Right, the power density of dDpMe^GABA^ ChR2 and dDpMe^GABA^ mCherry groups during different phases of laser stimulation from REM sleep (pre-stimulation, stimulation, and post-stimulation). **d** The probabilities of different brain states in GAD2-IRES-Cre mice after laser onset falling on REM sleep. The red lines indicate a statistical difference between the ChR2-mCherry and mCherry group (*F*_(1,637)(REM)_ = 301.10, *P* < 0.001; *F*_(1,637)(NREM)_ = 92.97, *P* < 0.001). **e** The pie charts and histograms show changes in brain states during laser stimulation initiated from different brain states (REM → NREM, *P* = 2.3 × 10^−12^; Wake→NREM, *P* = 7.9 × 10^−9^; ChR2-mCherry group, *n* = 5; mCherry group, *n* = 4). **f** Schematic of optogenetic experiments with EEG/EMG recordings of a GAD2-IRES-Cre mouse with Cre-dependent eNpHR-eYFP AAV injected into the dDpMe. Drawings of superimposed eYFP-expressing sites with the position of micropipette tips (*n* = 6) and typical fluorescent images in the dDpMe are shown, dorsomedial to the ChAT-labelled PPT (red). **g** All trials (*n* = 163) and the probabilities of different brain states from six mice before, during, and after constant 593-nm laser exposure. **h** Example recording of EEG power spectrograms, EEG/EMG traces, and hypnograms after bilateral laser stimulation above the dDpMe (593 nm, 60 s) in terms of NREM sleep. The power density of dDpMe^GABA^ eNpHR and dDpMe^GABA^ eYFP groups during different phases of laser onset falling on NREM sleep (pre-stimulation, stimulation, and post-stimulation). **i** The probabilities of different brain states in GAD2-IRES-Cre mice after laser onset falling on NREM sleep. The red lines indicate a significant difference between the eNpHR-eYFP or eYFP group (in NREM: *F*_(1,608)(REM)_ = 174.4, *P* < 0.001; *F*_(1,608)(NREM)_ = 130.2, *P* < 0.001). **j** Pie charts and histograms showing major states during laser stimulation initiated from different brain states, which were assessed by two-tailed *t*-tests: eNpHR-eYFP group, *P*_NREM→REM_ = 9.0 × 10^−6^; eNpHR-eYFP group, *n* = 6; eYFP group, *n* = 4. Data represent means ± SEM. *P < 0.05, **P < 0.001. The red or yellow horizontal lines in power density analysis indicate a statistical difference between pre-stimulation, stimulation, or post-stimulation (*P* < 0.05, red for pre-stimulation and stimulation; yellow for pre-stimulation and post-stimulation, two-way ANOVA with posthoc of the Sidak’s multiple comparisons test). DR dorsal raphe.

To investigate the roles of the dDpMe^GABA^ neurons in REM sleep, we applied targeted optogenetic stimulation via bilateral stereotaxic microinjections of AAV vectors carrying Cre-dependent ChR2-mCherry or mCherry^26,30^ into the dDpMe of GAD2-IRES-Cre mice (Fig. 2a and Supplementary Fig. S2a). Whole-cell recordings in brain slices revealed that ChR2-positive dDpMe^GABA^ neurons were depolarized with high temporal precision after 470-nm light stimulation (Supplementary Fig. S2b). Based on in vitro fidelity tests and in vivo latency comparisons, we selected 30 Hz as the light-stimulation frequency for subsequent experiments (Supplementary Fig. S2b). To quantify the effect, we aligned all trials from five mice by the time of laser stimulation. We found a sharp decrease in REM sleep, a rapid decrease within 10 s after laser onset, and a complementary increase in NREM sleep. In addition, REM sleep was found to rebound obviously after laser delivery (Fig. 2b). In detail, blue-light delivery in mice during REM sleep immediately terminated theta-dominated REM sleep, with a latency of 1.40 ± 0.20 s, and induced a rapid entry into the delta-dominated NREM sleep (Fig. 2c and Supplementary Fig. S2c). When REM and NREM bouts were quantified in 4 s epochs during light stimulation, REM sleep was dramatically decreased, and NREM sleep was significantly increased compared to that of the control group (93.5 ± 4.2%; Fig. 2d, e). Furthermore, light stimulation during wakefulness or NREM sleep was found to prolong NREM sleep and enhance wake-to-NREM transitions (Supplementary Fig. S2d–g). These findings demonstrated that dDpMe^GABA^ neuronal activation effectively promoted transitions from REM sleep or wakefulness to NREM sleep. Additionally, we found that the duration of the wake episodes appearing after light-induced REM sleep suppression was significantly decreased by 60.5% (*P* = 4.41 × 10^−3^), whereas the duration of NREM-sleep episodes after the suppression of REM sleep was increased by 82.43% (*P* = 0.032) (Supplementary Fig. S2h). Furthermore, we found that after dDpMe^GABA^ neuronal activation, the light-suppressed REM-sleep duration negatively correlated with re-occurrent REM-sleep duration, whereas the light-suppressed REM-sleep duration positively correlated with time intervals between REM cessation and re-occurrence (Supplementary Fig. S2i). Taken together, these findings demonstrated that the optogenetic activation of dDpMe^GABA^ neurons immediately terminated REM sleep and initiated NREM sleep as well as increasing wake-to-NREM transitions. Moreover, a prolonged re-occurrent REM-sleep duration was observed when the light stimulation was terminated.

### Inactivation of dDpMe^GABA^ neurons induced excessive REM sleep and promoted NREM-to-REM transitions

Next, we stereotaxically injected AAV constructs carrying Cre-dependent eNpHR (an inhibitory optogenetic protein) or eYFP (control) into the dDpMe of GAD2-IRES-Cre mice. We successfully labeled dDpMe^GABA^ neurons by immunohistochemistry and assessed their activity via patch-clamp recordings (Fig. 2f and Supplementary Fig. S3a, b). We aligned all trials from six mice with the timeline of laser stimulation and found fast induction of REM sleep within 20 s after laser onset and a complementary decrease in NREM sleep (Fig. 2g). In comparison with that in dDpMe^GABA^-eYFP mice, continuous yellow-light delivery for 60 s during NREM sleep effectively initiated REM sleep in dDpMe^GABA^-eNpHR mice with a significant increase in theta ratio (Fig. 2i, h, and Supplementary Fig. S3c). The latency to REM sleep induced by yellow-light delivery to dDpMe ^GABA^-eNpHR neurons was 18.49 ± 1.98 s (Supplementary Fig. S3c). The major-stage probability of REM sleep during yellow-light delivery was drastically increased from 6.4% in the control group to 61.1% in dDpMe^GABA^-eNpHR mice (Fig. 2j). In contrast, optogenetic inhibition of dDpMe^GABA^ neurons during REM sleep or wakefulness did not alter these behavioral stages (Supplementary Fig. S3d–g). Furthermore, we identified subsequent changes in the REM/NREM sleep cycle after yellow-light delivery. The durations of both REM sleep and wakefulness following optogenetic inhibition of dDpMe^GABA^ neurons were significantly increased (65.12% and 79.66%, respectively, *P* < 0.05; Supplementary Fig. S3h). Similar to dDpMe^GABA^ neuronal activation, we found that the duration of REM sleep induced by optogenetic inhibition of dDpMe^GABA^ neurons positively correlated with both re-occurrent REM sleep duration and REM-REM intervals (Supplementary Fig. S3i). These findings demonstrated that optogenetic inhibition of dDpMe^GABA^ neurons induced excessive REM sleep, suppressed NREM sleep, and increased the probability of NREM-to-REM transitions.

### Neural circuits of dDpMe GABAergic projections in regulating brain states

**Fig. 3:**
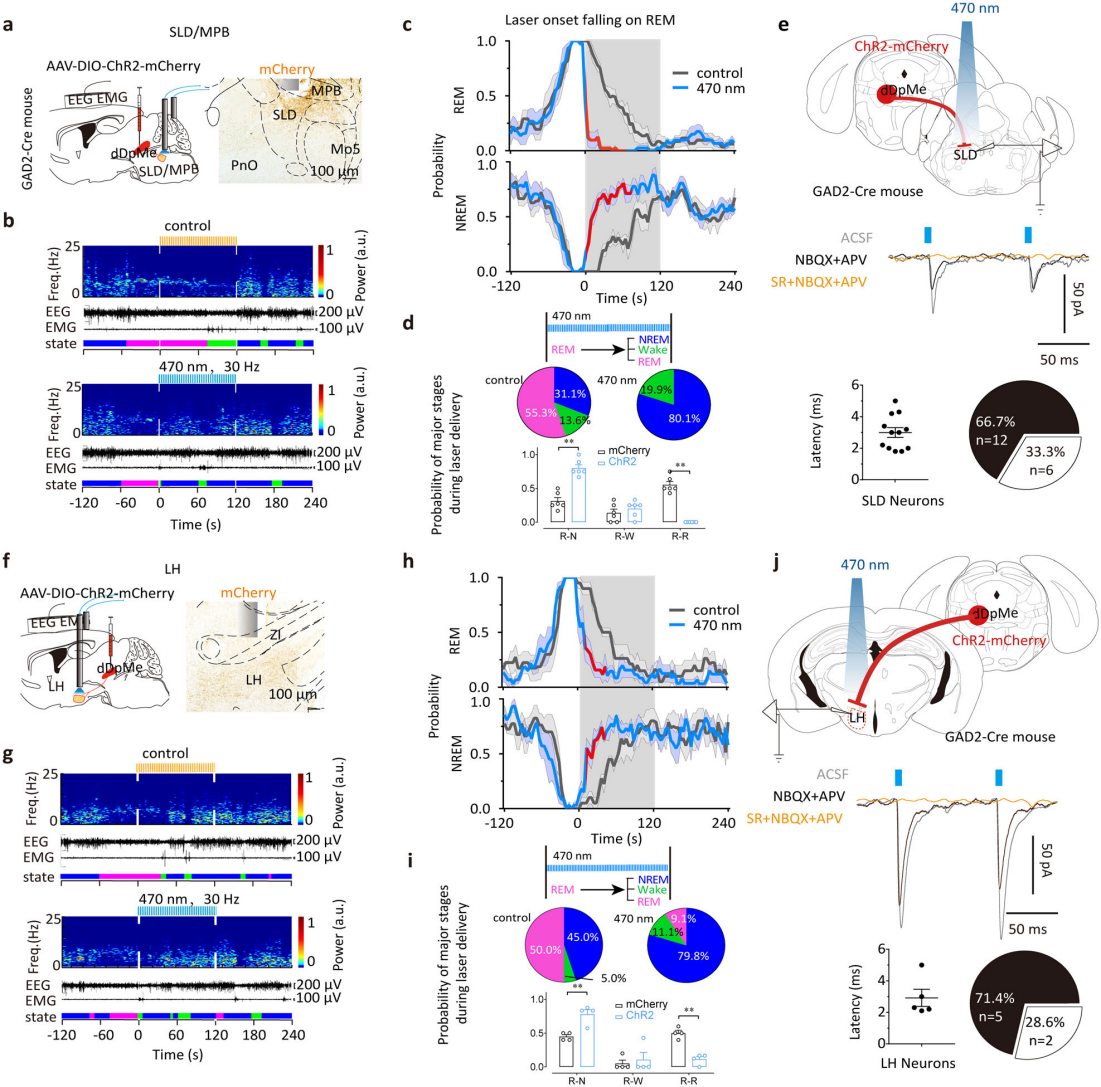
Activation of dDpMe^GABA^ axonal terminals in the SLD/MPB or LH suppresses REM sleep and promotes REM-to-NREM transitions. Schematic for optogenetic experiments and polygraphic recordings of the dDpMe-SLD/MPB (**a**) and dDpMe-LH pathway (**f**) with dDpMe neurons expressing ChR2-mCherry in GAD2-IRES-Cre mice. Example recordings of EEG power spectrograms, EEG/EMG traces, and hypnograms in GAD2-IRES-Cre mice after bilateral blue-light stimulation (bottom, 470 nm, 30 Hz, 5 ms, 120 s) or yellow-light stimulation (top, 593 nm, 30 Hz, 5 ms, 120 s) of dDpMe^GABA^ axonal terminals in the SLD/MPB (**b**) LH (**g**). Time courses of REM sleep (top) or NREM sleep (bottom) in GAD2-Cre mice after 593-nm or 470-nm laser stimulation of axonal terminals in the dDpMe or ChR2-mCherry-expressing neurons in the SLD/MPB (**c**), LH (**h**) initiated from REM sleep. The red lines indicate a significant difference (*P* < 0.05) compared to the control group, as assessed by two-way ANOVA (posthoc Sidak’s multiple comparison tests). *F*_(1,728)(REM)_ = 149.7, *P* < 0.001; *F*_(1,728)(NREM)_ = 123.8, *P* < 0.001; *F*_(1,728)(wake)_ = 0.69, *P* = 0.41 (c). *F*_(1,542)(REM)_ = 65.22, *P* < 0.001; *F*_(1,542)(NREM)_ = 10.98, *P* < 0.001; *F*_(1,524)(wake)_ = 25.43, *P* < 0.001(h). Pie charts and histograms showing major states during laser stimulation initiated from REM sleep (**d**) ChR2-dDpMe-SLD, *n* = 6; ChR2-dDpMe-LH (**i**), *n* = 4. Diagram showing the injection of AAV-ChR2 into the dDpMe of GAD2-Cre mice, and the response recorded in the SLD (**e**) or LH (**j**). Photo-stimulation (5 ms) evoked IPSCs in an SLD neuron, which was abolished by SR 95531 (a GABA_A_ antagonist). The latency of photo-stimulation-evoked IPSCs was counted from 12 responded SLD neurons in four mice (**e**) or 5 responded LH neurons in three mice (**j**). The mean latency of SLD neurons responding to the laser was 2.99 ± 0.31 ms (**e**) and that of LH neurons was 2.92 ± 0.54 ms (**j**). Number and proportion of recorded SLD or LH neurons showing a positive or negative response to the photo-stimulation of dDpMe neural terminals. Data represent means ± SEM. *P < 0.05, **P < 0.01. PnO oral part of pontine reticular nucleus, Mo5 motor trigeminal nucleus, ZI zona incerta.

We next sought to clarify the neural pathway for the conversion of REM sleep to NREM sleep by GABAergic neurons in the dDpMe. To this end, AAV-DIO-ChR2-mCherry constructs were injected into the dDpMe and optical fibers were inserted into the SLD/medial parabrachial nucleus (MPB), LH, or paraventricular thalamus (PVT) to activate the axonal terminals of projecting dDpMe^GABA^ neurons in GAD2-Cre mice (Fig. 3a–c, f–h and Supplementary Fig. S4a–d). The patch-clamp data showed that the light-evoked inhibitory postsynaptic potentials were completely blocked by SR95531, the GABA_A_ receptor antagonist, but not the AMPA and NMDA receptor antagonists NBQX and D-APV, indicating that dDpMe^GABA^ axonal activation mostly induced GABA release into SLD or LH neurons (Fig. 3e, j). Moreover, REM sleep was immediately blocked and NREM sleep was significantly increased in mice after optogenetic activation of the SLD/MPB or LH (Fig. 3c, h). In contrast, there was no effect in mice with activation of dDpMe^GABA^ axonal terminals in the PVT (Supplementary Fig. S4a–c). Interestingly, the probability of brain states showed that photostimulation of terminals in the SLD/MPB or LH of dDpMe^GABA^-ChR2 mice significantly decreased REM sleep by inducing an immediate transition from REM to NREM sleep, which was in contrast to the findings observed in mCherry-expressing control mice. The pie charts and histograms show that REM-sleep probability decreased and NREM-sleep probability increased during light stimulation in the SLD/MPB or LH regions, but not in the PVT (Fig. 3d, i and Supplementary Fig. S4d–e). These results indicate that dDpMe^GABA^ neurons suppressed REM sleep via the dDpMe-SLD/MPB and dDpMe-LH circuits.

### GABAergic dDpMe-Glutamatergic SLD neural pathway is essential for controlling REM-sleep termination and REM-to-NREM transitions

The onset of REM sleep is controlled by the nuclei of REM sleep promotion, such as the glutamatergic neurons in the SLD and GABAergic neurons in the ventral medulla^17^, which are essential for REM initiation and maintenance^12–14^. The SLD plays a critical role in controlling REM sleep and muscle atonia, and 84% of the REM active neurons are glutamatergic^13,31^. It has been reported that SLD glutamatergic (SLD^Glu^) neurons play a key role in generating REM sleep^20,32^. To test whether the SLD^Glu^ neurons received dDpMe inputs, we mapped the whole-brain inputs to SLD^Glu^ neurons via rabies viral (RV) retrograde tracing^33,34^ in the Vglut2-IRES-Cre mice (Supplementary Fig. S5a). The starter cells stained by yellow were co-infected with AAV helper viruses (GFP) and RV (DsRed), and the violet neurons presented ChAT immunoreactive signals for the laterodorsal tegmental nucleus and motor trigeminal nucleus, which were the border of the SLD (Supplementary Fig. S5b, c). To our surprise, we found that the dDpMe contributed the highest percentage of inputs to SLD glutamatergic neurons (9.11 ± 0.85%), whereas the vlPAG, LH, and lateral paragigantocellular nucleus contributed 3.08% ± 0.41%, 2.40% ± 0.21%, and 1.25% ± 0.34%, respectively (Supplementary Fig. S5d). A representative section revealed RV-DsRed-labeled presynaptic input neurons projecting to the SLD^Glu^ neurons intensively distributed in the dDpMe, and dDpMe neurons double-stained with *GAD1* mRNA and DsRed comprised 68.36% ± 3.95% of all dDpMe DsRed neurons (Supplementary Fig. S5e–g). These results indicate that dDpMe GABAergic (dDpMe^GABA^) neurons are one of the major sources of neural inputs to SLD^Glu^ neurons.

**Fig. 4:**
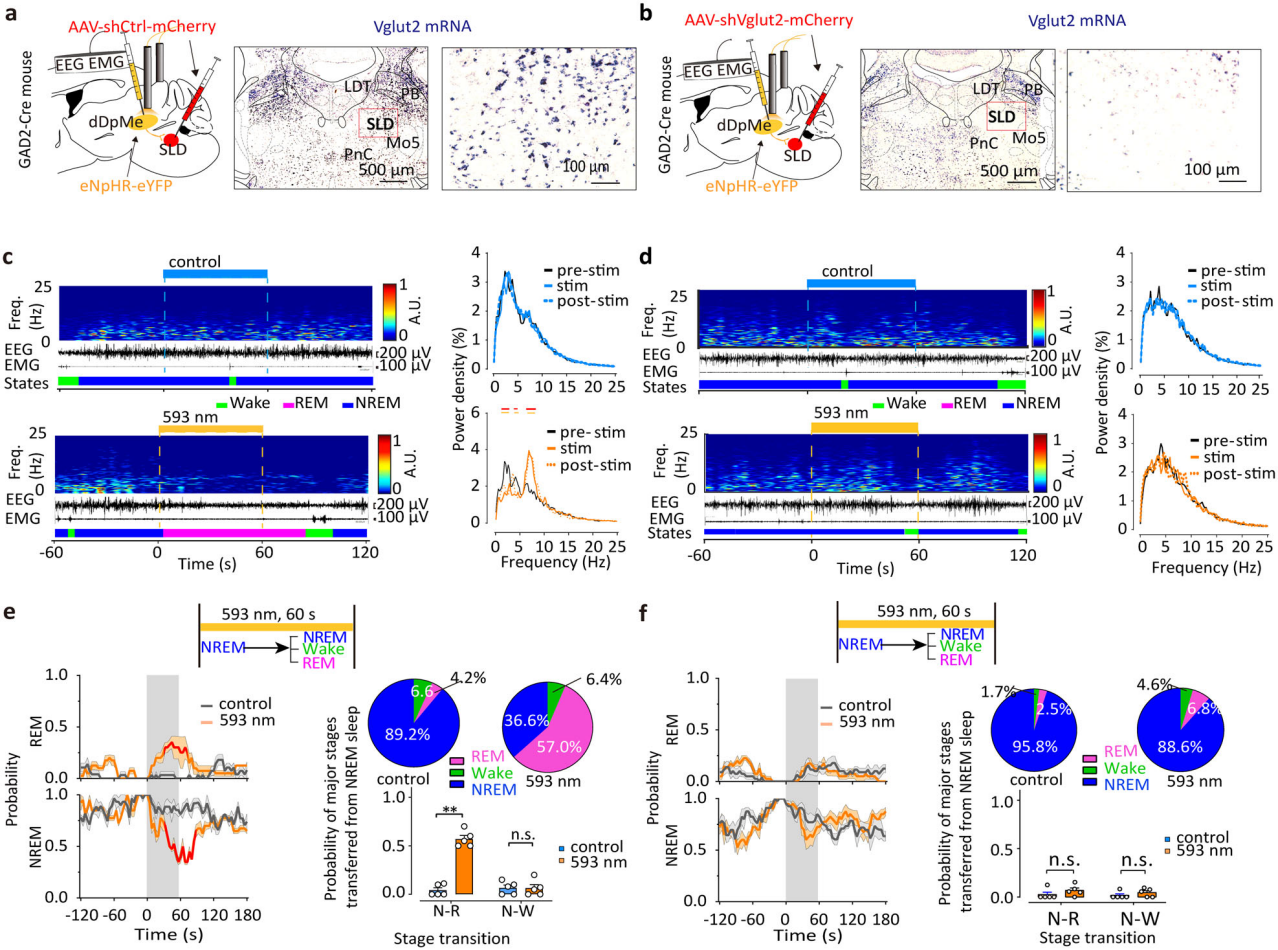
dDpMe^GABA^→SLD^Glu^ neural pathway is necessary for REM sleep termination and REM-to-NREM transitions. **a**, **b** Sagittal diagram for optical inhibition of the dDpMe by eNpHR after shRNA interference by shCtrl or shVglut2. shRNA interference of Vglut2 in the SLD was identified by in situ hybridization labelling *Vglut2*-mRNA. **c, d** Typical examples of EEG power spectrograms, EEG/EMG traces, and the mean percentage of power densities from GAD2-Cre mice with bilateral 593-nm laser delivery (bottom) or 470-nm laser delivery (top) to dDpMe eNpHR-expressing neurons. The horizontal bars indicate a significant difference (*P* < 0.01, two-way ANOVA with post-hoc Sidak’s multiple-comparison tests compared to 60-s pre-stimulation conditions; the red bars indicate pre-stimulation vs stimulation, and the orange bars represent pre-stimulation vs post-stimulation). Time courses of REM sleep (top) or NREM sleep (bottom) in GAD2-Cre mice with control or SLD^Glu^ interference after 593-nm or 470-nm laser stimulation of dDpMe eNpHR-eYFP-expressing neurons initiated from NREM sleep. The red lines indicate a significant difference (*P* < 0.05, two-way ANOVA with posthoc Sidak’s multiple comparison tests: shCtrl, *F*_(1,608)(REM)_ = 128.1, *P* < 0.001, *F*_(1,608)(NREM)_ = 232.5, *P* < 0.001; shVglut2, *F*_(1,608)(REM)_ = 0.56, *P* = 0.46, *F*_(1,608)(NREM)_ = 0.02, *P* = 0.89). **e**, **f** *P*ie chart and histograms showing major brain states during laser stimulation initiated from NREM sleep in the shCtrl group (**e**) or shVglut2 group (**f**), as assessed by two-way ANOVA with post-hoc Sidak’s multiple comparison tests: SLD-shCtrl group, *F*_(1,16)_ = 64.74, *P*_(NREM→REM)_ = 8.45 × 10^-9^; SLD-shVglut2 group, *F*_(1,16)_ = 2.71, *P*_(NREM→REM)_ = 0.33. shVglut2 group_,_ *n* = 5; shCtrl group, *n* = 5. Data represent means ± SEM. *P < 0.05, **P < 0.01. PnC caudal part of pontine reticular nucleus.

To determine whether SLD^Glu^ neurons are necessary for dDpMe^GABA^ neurons to control REM-to-NREM transitions, we used short-hairpin RNAs (shRNAs) targeting *Slc17a6* mRNA that encodes vesicular glutamate transporter 2 (Vglut2)^12,33^, to block synaptic glutamate release from SLD neurons. We verified the absence of *Vglut2* mRNA by in situ hybridization of brain sections from shVglut2-mice compared to *Vglut2* mRNA expression in shCtrl-injected mice (Fig. 4a, b, and Supplementary Fig. S6a). REM sleep was not induced by inhibition of dDpMe^GABA^ neurons after blocking glutamatergic neurotransmission in the SLD, while REM sleep was enhanced in dDpMe^GABA^-eNpHR/SLD shCtrl mice. Compared to blue-light stimulation (control), a shortened mean latency from 112.80 ± 3.27 s to 44.19 ± 8.24 s (Supplementary Fig. S6b) and increased probability of NREM-to-REM transitions from 4.2% to 56.1% were revealed in dDpMe^GABA^-eNpHR/SLD shCtrl mice after stimulation with yellow light (Fig. 4c, e). In contrast, yellow-light inhibition failed to enhance REM sleep in dDpMe^GABA^-eNpHR mice injected with shVglut2 into the SLD (Fig. 4d, f). In addition, we found that the mice with SLD glutamate release deficiency without manipulation of GABAergic neurons in the dDpMe showed a slight decrease in REM-sleep amounts over 24 h from 111.50 ± 9.68 s to 83.38 ± 5.27 s compared to control mice (*t* = 2.55, *P* = 0.03; Supplementary Fig. S6c), and atonia increased after shRNA inhibition of the SLD in mice (Supplementary Fig. S6d, e), which is consistent with Sara Valencia Garcia et al. reported in 2017^12^. These data demonstrated that the suppression of the dDpMe^GABA^–SLD^Glu^ pathway is responsible for initiating REM sleep.

Because AAV injections into the SLD inadvertently included a small portion of glutamatergic PB neurons, we specifically injected shVglut2 into the PB of dDpMe^GABA^-eNpHR mice (Supplementary Fig. S7a). However, we found that the inactivation of glutamatergic neurotransmission in the PB^Glu^ did not suppress REM-sleep induction by optogenetic inhibition of dDpMe^GABA^ neurons (Supplementary Fig. S7b, c), suggesting that the dDpMe^GABA^–PB^Glu^ pathway was not required for REM-sleep suppression.

Collectively, the data demonstrated that SLD^Glu^ neurons are essential for REM-sleep suppression of dDpMe^GABA^ neurons.

### dDpMe GABAergic neurons relieved cataplexy in orexin-neural-lesion mice

**Fig. 5:**
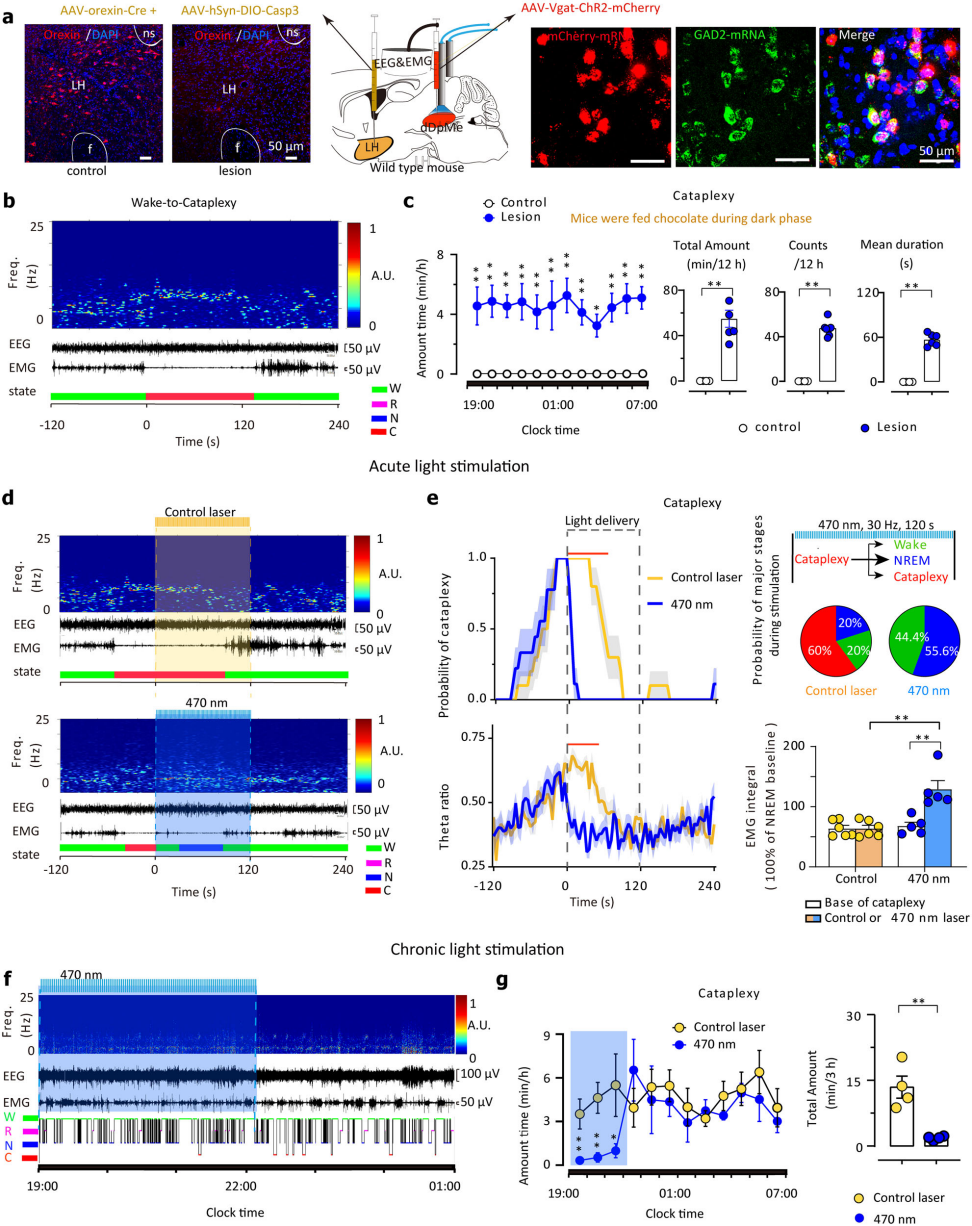
Activation of the dDpMe shuts down cataplexy attacks in mice with orexin neuronal lesions. **a** Schematic of a celltype-specific lesion combined with optogenetic stimulation and poly-graphic recordings of the dDpMe in mice. Left-most, orexin-stained neurons in the LH are present in the control group but absent in the caspase-3 lesion group. Right-most, Images of dDpMe AAV-Vgat-ChR2-mCherry neurons co-labelled with GAD2 mRNA. **b** Example recordings of a wake-to-cataplexy transition, which contains EEG power spectrograms, EEG/EMG traces, and hypnograms. **c** The time, total amount, counts, and mean duration of cataplexy in wild-type mice between the control group and lesion group over a 12-h night period. Regarding the amount of time, significant differences were assessed by two-way ANOVA for time course (posthoc Sidak’s multiple comparisons tests): lesion group vs control group, *F*_1, 72_ = 104.7, *P* < 0.001. The difference in total amount of 12-h night time, counts, and mean duration of cataplexy between the two groups were assessed by Student’s *t-test*, *P* < 0.001. **d** Example recordings of EEG power spectrograms, EEG/EMG traces, and hypnograms in wild-type mice, with bilateral blue-light stimulation (bottom, 470 nm, 30 Hz, 5 ms, 120 s) or yellow-light stimulation (top, 593 nm, 30 Hz, 5 ms, 120 s) of dDpMe^GABA^ neurons. **e** Time courses of cataplexy, theta ratio, the probability of major stage, and EMG integral in wild-type mice after 2 min control (593-nm) or 470-nm laser stimulation of dDpMe ChR2-mCherry-expressing neurons initiated from cataplexy. The red lines indicate a statistical difference between the ChR2-mCherry and mCherry groups (*P* < 0.01). EMG integral assessed by Student’s *t-test* (*P* < 0.01). **f** EEG power spectrograms, EEG/EMG traces, and hypnograms of wild-type mice with orexin neuronal lesions following the application of 3-h light stimulation (30 Hz, 5 s per 30 s) in dDpMe^GABA^ ChR2 neurons. **g** Difference in the time and total amount of cataplexy in wild-type mice between the control group and lesion group during 3-h light stimulation. Regarding the time, significant differences were assessed by two-way ANOVA for time course (post-hoc Sidak’s multiple comparisons tests): *lesion group vs control group, *F*_1, 72_ = 7.117, *P* = 0.0094. Differences in the total amount of each brain state during 3-h light stimulation were assessed by Student’s *t-test*. *P* = 0.0036. Data represent means ± SEM. *P < 0.05, **P < 0.01. ns nigrostriatal bundle, f forn.

To determine the roles of dDpMe GABAergic neurons in cataplexy, we applied optogenetic activation of dDpMe GABAergic neurons in conditional orexin-neural-lesion mice. We injected Vgat-ChR2-mCherry AAV viruses into the dDpMe to target GABAergic neurons, and injected orexin-Cre/dio-caspase-3 mixed AAV viruses into the LH area. Typical images showed orexin-immunoreactive LH area had no orexin expression after 2 weeks (Fig. 5a) and that AAV-GFP virus-infected neurons appeared like orexin-immunoreactive neurons (Supplementary Fig. S8a). Moreover, most of mCherry-labeled Vgat neurons were *GAD2* mRNA-positive and largely co-localized with GABA (Fig. 5a and Supplementary Fig. S8b). According to the previous studies, we defined the criteria for cataplexy as described in detail in Materials and Methods^35^. As the chocolate was commonly used as emotionally rewarding stimuli to trigger cataplexy in mice^36–38^, we fed the mice chocolate during the active period. We found that orexin-caspase-3 mice occurred cataplexy with sudden muscle weakness and REM-like sleep during wakefulness (Fig. 5b and Supplementary Video S1) and exhibited a high amount of cataplexy (*F*_(1,72)_ = 104.7, *P* < 0.001, ANOVA; Fig. 5c, left). The average amount of cataplexy during the dark period was 45.06 ± 4.30 min in orexin-neural-lesion mice, while the episode number and mean duration of cataplexies were 47.17 ± 3.01/12 h and 56.70 ± 3.37 s, respectively (Fig. 5c, right). Besides, wakefulness decreased accompanied by REM sleep increasing in the dark phase after lesion of orexin neurons (Supplementary Fig. S8c).

We next investigated whether cataplexy was regulated by dDpMe GABAergic neurons. We found that the cataplexy attacks were shut down after applying 470 nm-light stimulation in dDpMe GABAergic neurons for 120 s. In comparison with that in yellow-light control delivery, 30 Hz 470 nm-light delivery to Vgat-ChR2 neurons effectively and sharply curbed cataplexy attacks in orexin-caspase-3 mice (Fig. 5d and Supplementary Video S2). The probability of cataplexy during 470 nm delivery was drastically decreased and the theta ratio, the ratio of the power within the theta band (6–10 Hz) to the power within whole band, which is dominant in REM sleep and cataplexy (*F*_cataplexy(1,1548)_ = 115.50, *P* < 0.001; *F*_theta(1,1638)_ = 26.22, *P* < 0.001, two-way ANOVA; Fig. 5e, left), and the probability of NREM sleep and wakefulness increased correspondingly (Supplementary Fig. S9a). The pie chart clearly showed that after 470 nm light delivery, cataplexy was abolished and NREM sleep almost tripled compared to control light stimulation. Moreover, the EMG integral during 470 nm light delivery in cataplexy divided by the baseline during NREM sleep was increased significantly compared to that during 593 nm light delivery or baseline in cataplexy (*F*_EMG(3.18)_ = 14.17, *P* < 0.001; Fig. 5e, right). To investigate the long-lasting effect of dDpMe GABAergic neurons on cataplexy, we applied 30 Hz 470 nm, 5 ms, 5 s per 30 s light delivery in dDpMe GABAergic neurons for 3 h after feeding the mice with chocolate. The amount of cataplexy per hour was decreased significantly (*F*_(1,72)_ = 7.12, *P* = 0.0094; Fig. 5f, g), and the total amount of cataplexy for 3 h at 470 nm delivery was decreased significantly from 13.85 ± 2.58 min to 1.88 ± 0.24 min (*t* = 4.61, *P* = 0.0036, unpaired *t-test*; Fig. 5g). At the same time, the time course or total amount of NREM, REM, and wakefulness did not change significantly during or after light stimulation (Supplementary Fig. S9b). Therefore, we reveal that dDpMe GABAergic neurons play an important role in inhibiting cataplexy attacks.

Furthermore, to investigate whether the suppression of the cataplexy is directly from the projection to the LH or other brain regions, we applied optogenetics to stimulate the downstream of dDpMe^GABA^ neurons in the cataplexy model (Supplementary Fig. S10). As a result, we found that not only dDpMe^GABA^-LH pathway but also the dDpMe^GABA^-SLD pathway inhibited cataplexy.

Taken together, these findings show that the dDpMe^GABA^-SLD^Glu^ neural circuit is essential for REM sleep termination and REM-to-NREM sleep transition, and that dDpMe^GABA^ neurons are vital to relieve cataplexy attacks (Supplementary Fig. S11).

## Discussion

The present findings revealed that substantial dDpMe^GABA^ neurons are REM relatively inactive and the activity changed across state transitions, providing strong evidence that dDpMe^GABA^ neurons control REM-sleep termination and promote REM-to-NREM transitions as well as increasing wake-to-NREM transitions via SLD glutamatergic and LH neurons. We built a rodent cataplexy model, which mimicked the cataplexy attacks observed in the transgenetic orexin-KO mouse model established by Chemelli et al. in 1999^39^. We demonstrated that celltype-specific manipulation of dDpMe inhibitory neurons abolished cataplexy attacks in orexin neural lesion mice. Furthermore, we also revealed that REM-sleep induction can occur within seconds via dDpMe^GABA^ neuronal inhibition.

As previous data have shown, there are REM relatively active neurons and REM relatively inactive neurons in the laterodorsal tegmental nucleus^40^, pedunculopontine tegmental nucleus^41,42^ or vlPAG^19^. Comparing the neurons in these brainstem nuclei, the population activity of dDpMe^GABA^ neurons was found to be REM relatively inactive by fiber photometry recording of calcium signals. By spike sorting, we identified that dDpMe^GABA^ neurons comprise Type1 and Type2 subpopulations. The in vivo optical tetrode recording in combination with the Cre-loxp system was effective for identifying the cell-type-specific neurons. The method to acquire the activity of specific neurons was faster and more precise than that involve analyzing discharge characteristics of neurons used in previous studies^43^. Based on in vivo optical tetrode recording, we found Type 2 neurons are more active during REM sleep, and the activity of Type1 neurons is lowest in REM sleep and highest in wakefulness. Combining with population activity of dDpMe neurons by fiber photometry, Type 1 neurons possibly occupy dominantly the population in dDpMe. We suppose the Type 2 neurons are more likely to inhibit REM-promoting neurons (such as the SLD neurons) constantly during REM sleep whereas Type 1 neurons are supposed to inhibit REM-promoting neurons just before REM to other states.

By applying virus-based tracing and optogenetics, we identified the neural circuits of the dDpMe, which may be useful to establish their physiological and pathological function in the future. Genetically engineered RV is widely used in neuroscience for its cell-type-specific infection of neurons, the fact that it does not affect passing neuronal tracts, and its effective passage across the known synaptic connections^44^. Accordingly, using RV-mediated retrograde tracing in Vglut2-IRES-Cre mice, we demonstrated that the dDpMe is the most dominate inputs to SLD glutamatergic neurons. This result was consistent with previous non-neuronal selective findings using cholera toxin subunit B retrograde tracing^22^. These studies may indicate that the dDpMe GABAergic neurons play a key role in SLD neuronal-related functions, such as the switch of REM sleep and REM sleep behavior disorders. Besides the dDpMe^GABA–^SLD^Glu^ circuit, we showed that the dDpMe^GABA^-LH circuit is crucial to REM-sleep control. It has been proposed that the LH modulates REM sleep by REM-sleep-promoting melanin-concentrating hormone (MCH) neurons and wake-promoting orexin/hypocretin neurons^15,45^. Indeed, Kroeger et al. demonstrated the importance of MCH neurons to dDpMe/vlPAG pathways for regulating REM sleep^46^. In this study, we identified dDpMe–LH pathways that participated in REM-sleep control, suggesting that dDpMe^GABA^ neurons send neuronal inputs to LH REM-promoting neurons, such as MCH neurons, to suppress REM sleep and cause alternative behavioral states. As the LH is the key node of substantial physiological and emotional activity, the bidirectional pathways of dDpMe–LH may be involved in regulating anxiety, stress, and insomnia.

Our work demonstrates a novel neuronal mechanism overlapping REM sleep and cataplexy inhibition, which was crucial for understanding the dysfunctional cycling of sleep stages and narcolepsy. In narcolepsy, owing to the lack of excitatory orexin inputs to the REM-inhibitory system of the brainstem (dDpMe, LC, vlPAG, etc.), positive emotions activate the limbic system, which inhibits the REM-inhibitory system and disinhibits the descending inhibitory systems during cataplexy. Systemic pharmacological medicines exist for treating cataplexy, such as tricyclic antidepressants, including protriptyline and clomipramine, but their anticholinergic side effects have limited their application^47^. Nowadays, sodium oxybate is considered as one of the most effective drugs for severe cataplexy, whereas its precise mechanism of action remains obscure^48^. Our work showed that when orexin neurons were damaged (orexin-caspase-3 lesion) and positive emotions were introduced (chocolate), activation of dDpMe GABAergic neurons still strongly inhibited cataplexy. The results revealed that dDpMe GABAergic neurons play vital roles in cataplexy. The ability of GABAergic neurons in the dDpMe to control REM-to-NREM transitions and relieve cataplexy might facilitate future research on the treatment of narcolepsy. Based on the evidence of Anaclet et al. 2011^49^ and Zoltan et al. 2019^31^, the SLD probably is the downstream of dDpMe GABAergic neurons for relieving cataplexy. When orexinergic excitatory inputs to the dDpMe was interrupted by orexin lesion in the cataplexy model, the GABAergic projects to SLD will be inactive, which lead the SLD to be disinhibited and induce cataplexy. When dDpMe GABAergic neurons were activated, the hyper-excited SLD will be inhibited and cataplexy attacks were curbed immediately. On the other hand, Naganuma et al. in 2018 found that MCH neurons contribute to dysregulation of REM sleep in narcolepsy^50^, suggesting that the LH might also be the downstream of dDpMe GABAergic neurons for relieving cataplexy. In our study, we proved that both of dDpMe^GABA^–LH and dDpMe^GABA^–SLD pathways inhibited cataplexy in orexin lesion mice.

In conclusion, we revealed that the neural activity of dDpMe^GABA^ neurons was firmly fixed with brain states. We also identified an essential role of dDpMe^GABA^ neurons in REM sleep termination and REM-to-NREM sleep transitions, primarily through dDpMe^GABA^–SLD/LH neural circuits. Moreover, we uncovered that dDpMe^GABA^ neurons represent a vital switch in cataplexy, which would provide a novel target for the treatment of narcolepsy in the future.

## Materials and methods

### Mice

Optogenetic manipulations, fiber photometry, in vivo multichannel recording, and RNA-inference experiments were performed in male GAD2-IRES-Cre mice (Jackson Laboratory stock 010802) or male Vglut2-IRES-Cre mice (Jackson Laboratory stock 016963). Descriptions in detail are in Supplementary methods.

### Viral vectors

Viral vectors used for retrograde tracing and behavioral experiments were packaged by BrainVTA Co., Ltd. (Wuhan, China) or Tailtool Bioscience Co., Ltd. (Shanghai, China). The titer of the EnvA-pseudotyped, glycoprotein (RG)-deleted, and DsRed-expressing rabies virus (RV-EnvA-ΔRG-DsRed, Brain VTA, # R01002) was ~2 × 10^8^ infecting units/mL. The two adeno-associated virus (AAV) vectors, namely, AAV-EF1α-DIO-TVA-GFP (Brain VTA, # PT-0165) and AAV-EF1α-DIO-RV-G (Brain VTA, # PT-0023), were packaged into the 2/9 AAV serotype and titrated at ~3 × 10^12^ genome copies/mL^51–53^. The titer of the AAV-EF1α-DIO-ChR2-mCherry (Taitool Bioscience, # S0170-9), AAV-EF1α-DIO-mCherry (Taitool Bioscience, # S0312-9), AAV-EF1α-DIO-eNpHR-eYFP (Taitool Bioscience, # S0178-9), AAV-EF1α-DIO-eYFP (Taitool Bioscience, # S0320-9), AAV-Vgat-ChR2-mCherry (Brain VTA, # PT-0643), AAV-Orexin-Cre (promoter sequence designed from *Mus musculus hypocretin* (*Hcrt*), NM_010410, 1513 bp, Brain VTA, # PT-1573), AAV-EF1α-DIO-taCap3-TEVp (Taitool Bioscience, # S0236-9), and AAV-EF1α-DIO-eGFP (Taitool Bioscience, # S0270-9) was ~3–5 × 10^12^ infecting units/mL. Yoan Cherasse kindly provided the AAV-shVglut2-mCherry (5.6 × 10^12^ particles/mL) and AAV-shCtrl-mCherry (4.5 × 10^12^ particles/mL) vectors^12,54^.

### Surgeries and viral injections

Adult mice were anesthetized with isoflurane (5% induction, 1.5% maintenance) and placed in a stereotaxic apparatus (RWD Life Science, Shenzhen, China). Using a compressed air delivery system as previously described^55^, AAVs were slowly bilaterally microinjected (20 nL/min, the total volume of AAV the authors injected was 40–80 nL) into the dDpMe (AP: − 4.2 mm, ML: + 1.0 mm, DV: − 3.5 mm)^51^, SLD, AP: − 5.1 mm, ML: + 0.8 mm, DV: − 4.1 mm, or LH, AP: − 0.7 mm, ML: + 1.0 mm, DV: − 5.3 mm through a glass pipette. The glass pipette was left in place for an additional 20 min and was then slowly withdrawn. After three weeks, the mice used for in vivo studies were chronically implanted with EEG/EMG electrodes and fibers, the details are in Supplementary methods.

### Vigilance state assessment

The vigilance states were automatically scored offline by SleepSign (Kissei Comtec, Nagano, Japan) into 4-s-epoch wake, NREM, REM sleep, or cataplexy according to standard criteria based on EEG and EMG waveforms. The states were examined visually and manually corrected if necessary. We defined wakefulness as desynchronized EEG and high levels of EMG activity, NREM sleep as synchronized, high-amplitude, low-frequency (0.5–4 Hz) EEG signals in the absence of motor activity, and REM sleep as having pronounced theta-like (6–10 Hz) EEG activity and muscle atonia^56^. Cataplexy was defined by muscle atonia lasting more than 10 s after more than 40 s of wakefulness, and REM-like theta activity in the EEG^35^.

### Optogenetic stimulation

We performed optogenetic experiments during 12:00 p.m.-00:00 a.m. 3–4 weeks after injection of AAV-expressing eNpHR or ChR2. The optical fiber cannulas were attached to a rotating joint (FRJ_FC-FC, Doric Lenses, Canada) to relieve torque. The joint was connected via a fiber to a 473-nm blue laser or a 590-nm yellow laser, and the light pulses were generated through a stimulator (Master-8, A.M.P.I., Israel). For optogenetic activation of GABAergic dDpMe neurons or their axonal terminals in normal mice, each trial consisted of a 1-30-Hz pulse train lasting for 120 s using a 473-nm blue laser during inactive period (6–8 mW at the fiber tip, Shanghai Optogenetic stimulation in vivo Laser, China). For optogenetic inhibition of dDpMe^GABA^ neurons in normal mice, each trial consisted of continuous light lasting for 60 s using a 590-nm yellow laser during inactive period (6–8 mW at the fiber tip, Shanghai Optogenetic stimulation in vivo Laser, China). The inter-trial interval of optogenetic stimulation was more than 10 min. For optogenetic activation of GABAergic dDpMe neurons or their axonal terminals in orexin neuronal lesion mice, each trial of acute stimulation experiments consisted of a 30-Hz pulse train lasting for 120 s using a 473-nm blue laser during active period, whereas chronic stimulation experiments were performed by 30-Hz pulse train (5 s per 30 s) lasting for 3 h (6–8 mW at the fiber tip, Shanghai Optogenetic stimulation in vivo Laser, China).

### Fiber photometry

Following AAV-EF1α-DIO-GCaMP6f virus injection, an optical fiber (125 μm O.D., 0.37 numerical aperture (NA); Newdoon, Shanghai) was placed in a ceramic ferrule and inserted toward the dDpMe. Following the 2-week recovery period from the virus injection and implantation surgery, fiber-photometry experiments were performed as previously described^27,57–59^. The photometry analog voltage signals were digitized at 512 Hz and recorded by a Power 1401 digitizer and Spike2 software (CED, Cambridge, United Kingdom) simultaneously with polysomnographic recordings. The details of data analysis are in Supplementary methods.

### Multichannel recording by optic tetrodes

To record the activity of dDpMe GABAergic neurons, we used a custom-built optode, consisting of an optical fiber (0.2-mm diameter) surrounded by 16 microwire electrodes (13 μm, Sandivik, Pt/Ir F562, USA) twisted into tetrodes. The optical fiber and electrodes were inserted into a screw-driven microdrive. The optode was slowly lowered in 25–50 μm steps to search for light-responsive neurons. The multichannel signals were digitized at 40 kHz and recorded by a 16-channel Plexon system and Omniplex software (Plexon, USA) simultaneously with polysomnographic recordings (digitized at 1 kHz). At the end of the experiment, electrolytic lesions were made by passing a current (100 mA, 10 s) through one or two electrodes to identify the end of the recording tract^60^. The details in sorting and analysis are in Supplementary methods.

### Statistical analysis

Data were assessed using one-way or two-way repeated-measures ANOVAs followed by Sidak’s multiple-comparison tests. When appropriate, Student’s *t*-tests were used to determine statistical significance of pre-planned comparisons. Data are expressed as the means ± standard error of mean (SEM). The linear relationship of the sleep duration and interval was measured by the Pearson product-moment correlation coefficient. A *P*-value < 0.05 was considered statistically significant.

The details of retrograde tracing, electrophysiological experiments in vitro, immunohistochemistry, immunofluorescence, and in situ hybridization are in Supplementary methods.

## Supplementary information

SUPPLEMENTAL MATERIAL

Video 1

Video 2

## Data Availability

The data that support the findings of this study are available from the corresponding author upon reasonable request.
