# Supplementary material for: A cluster of mesopontine GABAergic neurons suppresses REM sleep and curbs cataplexy
Source: Cell Discov. 2022 Oct 25;8:115. doi: 10.1038/s41421-022-00456-5 (PMC9592589; doi:10.1038/s41421-022-00456-5)
Supplement: Supplementary file 1 — SUPPLEMENTAL MATERIAL [file 41421_2022_456_MOESM1_ESM.pdf]

Supplementary Information for

A cluster of mesopontine GABAergic neurons suppresses REM sleep and curbs cataplexy

Ze-Ka Chen, Hui Dong, Cheng-Wei Liu, Wen-Ying Liu, Ya-Nan Zhao, Wei Xu, Xiao Sun, Yan-Yu Xiong, Yuan-Yuan Liu, Xiang-Shan Yuan, Bing Wang, Michael Lazarus, Yoan Chérasse, Ya-Dong Li, Fang Han, Wei-Min Qu, Feng-Fei Ding, and Zhi-Li Huang

Zhi-Li Huang & Feng-Fei Ding & Wei-Min Qu

Email: huangzl@fudan.edu.cn & francesding2016@163.com & quweimin@fudan.edu.cn

This PDF file includes:

Supplementary methods

Figures S1 to S11

Legends for Movies S1 to S2

Other supplementary materials for this manuscript include the following:

Supplementary Video S1 to S2

Supplementary methods

Mice

Male mice (25 ± 3 g) were bred and housed under an automatically controlled 12/12-h light/dark cycle (lights on at 07:00). After surgery, mice were housed for at least 1 week before further experiments. All of the procedures were performed in accordance with the recommendations of the China Regulations on the Administration of Laboratory Animals (the Decree NO.2 of National Science and Technology Commission of the People's Republic of China) and were approved by the Medical Experimental Animal Administrative Committee of the School of Basic Medical Sciences (Permit number: 20140226–024) at Fudan University (Shanghai, China).

Surgeries of implanting fibers and electrodes

Three weeks after injection, the mice used for *in vivo* studies were chronically implanted with EEG/EMG electrodes for polysomnographic recordings under isoflurane anesthesia (5% induction, 1.5% maintenance). The electrodes consisted of two stainless steel screws connected to EEG Teflon-coated wires, which were inserted through the skull, and two EMG Teflon-coated wires, which were bilaterally placed into both trapezius muscles. All of the electrodes were fixed to the skull with dental cement and attached to a microconnector [1]. For fiber photometry, the tips of fiber-optic cannulas (200-μm diameter; Newton Inc., Hangzhou, China) were inserted into the viral-injection sites before implantation of the electrodes. For optogenetic experiments, the tips of fiber-optic cannulas were inserted at 0.2 mm above the viral-injection sites or axonal-terminal locations before implantation of the EEG/EMG electrodes. The scalp wound was closed with surgical sutures, and each mouse was kept in a warm environment until it resumed normal activity as previously described [1]. After surgery, the mice were housed separately for approximately 7 days for recovery.

Polysomnography

The mice were first connected to the sleep recording equipment to habituate for 72 h before the formal video-polysomnography recordings. A cable with a slip ring (Kissei Comtec Co., Ltd, Japan) was connected to mice in the cage for 7 days before the initiation of EEG and EMG recordings. The EEG/EMG signals were amplified, filtered, and digitized as previously described [2].

RV-based retrograde tracing and cell counting

An RG-deleted RV strategy for retrograde tracing has been reported to mark monosynaptic inputs to specifically selected starter cells. This method has been successfully used in previous studies by our group. For cell mapping of neurons, neuronal somata were quantified semi-automatically via ImageJ software as previously described [3]. We divided each brain into six general structures, which together encompassed more than 50 specific brain regions containing all DsRed-labeled neurons throughout the brain (n = 4 mice).

Data analysis of Fiber photometry

Photometry data were exported to Matlab Mat files from Spike2, and then analyzed by customized Matlab software (Matlab, 2016a, MathWorks, United States) as described in our previous study. For each session,

the photometry signal F was converted to ΔF/F by calculating $\Delta F/F = (F - F_{mean})/F_{mean}$, where F_{mean} is the average fluorescence signal in each recording episode. For the analysis of mean fluorescence signal, we derived the value of the photometry signal by calculating the Z-score = $(F - F_{mean})/\sigma$, where σ is the standard deviation of the fluorescence signal. For the sleep-wake analysis, we recorded data for 4–6 h per mouse each day and calculated the averaged Z-score during all vigilance states. To analyze the state transition, we determined each state transition and aligned the Z-score in a ± 30 s window before and after the switch point.

Multichannel recording by optic tetrodes

The tetrode wire tips were plated with gold (Cyanida Gold solution, SIFCO Selective Plating) to adjust the impedance to 500–800 kΩ. The wire tips were 0.5 mm longer than the optical fiber end to achieve efficient photostimulation of the recorded neurons in vivo. For implantation, the electrode was slowly advanced into the dDpMe, controlled by a piezoelectric micromanipulator (Scientifica).

Spikes were sorted offline on the basis of the waveform energy and the first three principal components of a spike waveform on each tetrode channel. Single units were identified using the built-in principal component analysis in Offline Sorter software (Plexon, USA). The quality of each unit was assessed by the presence of a refractory period and quantified using the isolation distance and L-ratio. Units with an isolation distance < 20 or L_{ratio}> 0.1 were discarded.

To identify ChR2-tagged neurons, laser pulse trains (20 Hz or 40 Hz, with a duration of 1 and 0.5 s, respectively) were delivered every 1 or 2 min. A unit was identified as GABAergic if spikes were evoked by laser pulses at short first-spike latency (<8 ms) and the waveforms of the laser-evoked and spontaneous spikes were highly similar. Spikes during the laser pulse trains were excluded to compute the mean firing rate of each neuron in each brain state.

Data analysis of optogenetic experiments

To test whether the probability of sleep has changed after manipulation of these neurons' activity, we quantify the probability of stages transition from an identified initial state to the following dominated states. The following dominated state is defined as major stages during stimulation, which meant the phase with the largest proportion during total light delivery. For instance, if an initial phase of a trial is REM sleep, the major state is NREM sleep during stimulation, then we define this trial as a REM-NREM sleep transition. If in a mouse, there're totally 20 trials of initial phase of REM sleep, including 10 trials of REM-NREM transitions (R-N), 10 trials of REM-wakefulness transitions (R-W), 0 trial of REM sleep maintenance (R-R), we calculated the probability of major stage during stimulation is 50%(R-N), 50%(R-W), and 0%(R-R). Then, we calculated the mean probability of major stage by averaging the data of all mice.

Slice recordings

For slice preparations, male GAD2-IRES-Cre mice (6–8 weeks old) were microinjected with AAV containing optogenetic elements into the dDpMe under deep anesthesia. We provided at least 3 weeks for recovery and sufficient expression of viral constructs in the targeted somata and axonal terminals. Decapitation was performed rapidly after intracardial perfusion with 25 ml of ice-cold (4°C) oxygenated modified artificial cerebrospinal fluid (ACSF). The brain was then carefully harvested fixed onto a plate covered with the same solution as above. The composition of the modified ACSF was as follows (in mM): 213 sucrose, 10 glucose,

2.5 KCl, 1.25 NaH_2PO_4, 26 $NaHCO_3$, 3 $MgSO_4$, 0.1 $CaCl_2$, and 0.4 ascorbic acid. Coronal sections (280 μm) were cut on a vibratome (VT-1200, Leica Microsystems), and only those including targeted brain nuclei were selected and incubated in ACSF (32°C) for 30 min. The composition of the ACSF was as follows (in mM): 25 glucose, 119 NaCl, 2.5 KCl, 1.25 NaH_2PO_4, 26 $NaHCO_3$, 1 $MgCl_2$, and 2 $CaCl_2$ (at 32°C). All solutions were made fresh and were saturated with 95% O_2 and 5% CO_2 before use.

For whole-cell recordings, slices were placed in the recording chamber after 30-min recovery, and were perfused with oxygenated ACSF (30–32°C, 1–2 mL/min). Only healthy neurons were recorded, as confirmed by infrared differential-interference-contrast and fluorescence microscopy. In the current-clamp mode, we acquired the electrophysiological properties of the recorded neurons, including the resting membrane potential and action potentials. In the voltage-clamp mode, we held the potential at −70 mV for whole-cell recordings. Somata expressing ChR2-mCherry were stimulated with blue light (5-ms pulses of 1–2 mW, 470-nm light delivery via an LED through a X40 microscope objective). Somata expressing eNpHR-eYFP were stimulated with yellow light (1–2 mW, 593-nm continuous light delivery via an LED through a X40 microscope objective).

Data were collected, filtered, and digitized via pClamp 10.3 software (Axon Ins., USA), a MultiClamp 700B Microelectrode Amplifier (Axon Ins., USA), and Micro1401 (CED Ins., USA). Any recorded neurons with a R_a > 30 MΩ or a floating range > 20% were excluded from further analysis. All data were low-pass filtered at 1 kHz and digitized at 4 kHz.

Immunohistochemistry and immunofluorescence

Adult mice were deeply anesthetized and transcardially perfused with cold normal saline followed by 4% paraformaldehyde in 0.1 M phosphate-buffered saline (PBS). The brain was post-fixed for 2 h and then cryoprotected in 30% sucrose. After embedding in optimal-cutting-temperature compound, the brain was sectioned coronally at 30 μm on a freezing microtome (CM 1950, Leica). Sections were rinsed with 0.3% Triton-X in 0.01-M PBS and blocked with 10% normal bovine serum for 1 h. The sections were incubated with primary antibodies (goat anti-ChAT, 1:1000, Millipore AB143; rabbit anti-mCherry, 1:3000, Clotech 632496; rabbit anti-GABA, 1:1000, Thermofisher PA5-32241; polyclonal goat antibody against orexin A, 1:2000, SCB sc8070) in 0.01 M PBS for 12 h at 4°C. For immunofluorescence, sections were rinsed and incubated with an Alexa Fluor-conjugated IgG antibody (Invitrogen) for 1 h at room temperature. For immunohistochemistry, brain sections were incubated in the following: (1) a biotinylated goat anti-rabbit IgG solution (1:1000; Vector Laboratories BA-1000) for 90 min at room temperature; and (2) an ABC-HRP solution (1:1000; Elite kit, Vector Laboratories, PK-6100) for 60 min at room temperature. Finally, the sections were immersed in DAB Substrate Kit (DAB, Sigma-Aldrich, St. Louis, MO, USA). Three 10-min washes in PBS were performed between each step.

***In situ* hybridization**

In situ hybridization, with either glutamate decarboxylase 1 (GAD1, 67 kDa) or Vglut2 mRNA, was performed via digoxigenin riboprobes in brain sections. The brain sections were placed onto slides and were surrounded by water-repellent traces. Subsequently, the slides were post-fixed in 4% PFA for 20 min. *In situ* hybridization was processed as previously described [4,5]. DNA templates for the *in situ* hybridization probes were obtained by PCR from either wild-type-embryo or P0-mouse cDNA libraries. All of the buffers contained 0.1% RNase

inhibitor (diethyl pyrocarbonate, DEPC, B600154, Sangon Biotech). Finally, the sections were mounted on slides, dried, and coverslipped with Vectamount (Vector Laboratories). Images were acquired using a Leica confocal system or an Olympus IX71 microscope.

For DsRed immunohistochemistry combined with GAD1 or Vglut2 mRNA *in situ* hybridization, brain sections were placed onto slides and *in situ* hybridization was performed following completion of DsRed immunohistochemistry. All of the buffers contained 0.1% of RNase inhibitor.

Fluorescence *in situ* hybridization (FISH) was performed using RNAscope Multiplex Fluorescent v2 Assays according to the manufacturer's instructions (Advanced Cell Diagnostics). Cells with more than 10 fluorescent dots in the cytoplasm were considered to be positively labeled.

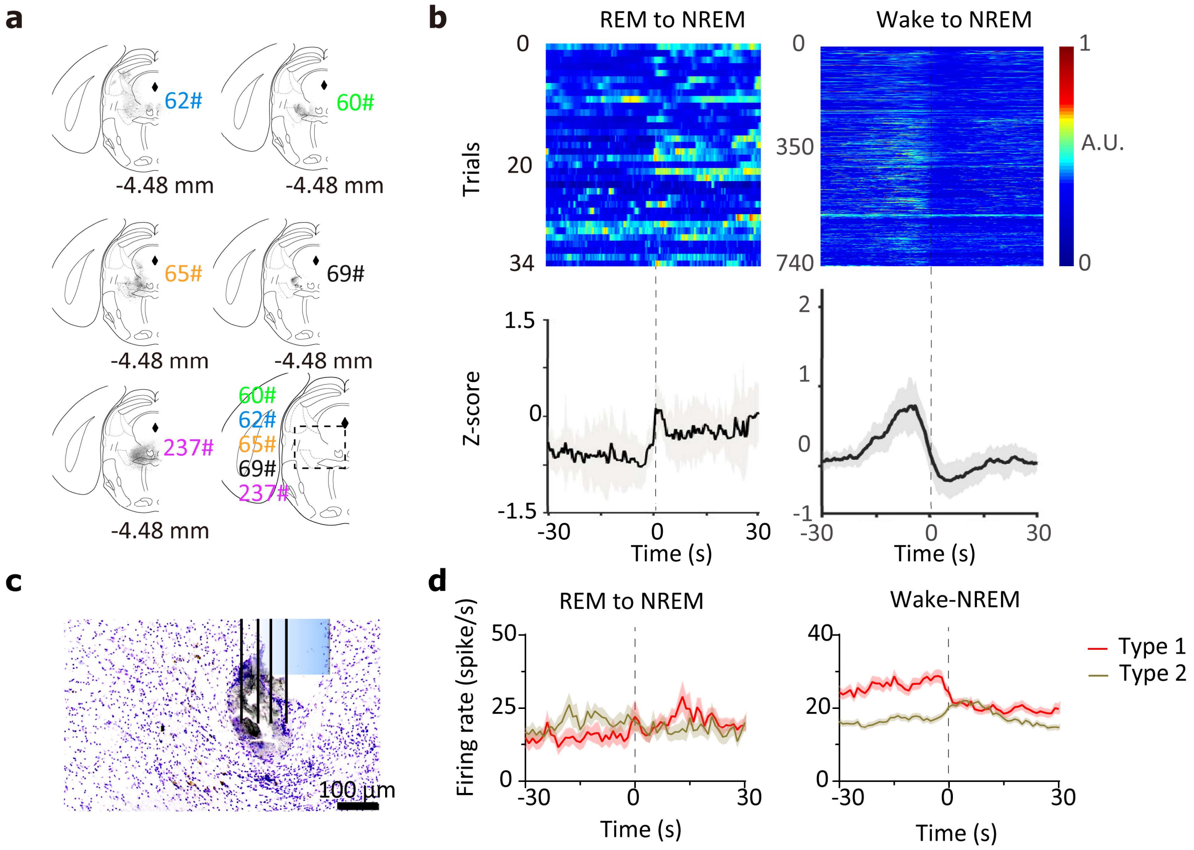

S1 Spontaneous dDpMe GABAergic neuronal activity in sleep-wake cycle by fiber photometry and multichannel recording *in vivo*.

(**a**), Coronal sections showing the distribution of GCaMP6f-expressed neurons and optic fiber tips in the dDpMe of each GAD2-IRES-Cre mice. (**b**), Ca^{2+} signals associated with transitions among NREM sleep and wakefulness. Top, individual transitions with color-coded fluorescence intensity. Bottom, Z-score of fluorescence changes from all the transitions expressed as mean (blue trace) ± SEM. (shading). (**c**), Recording sites of multichannel recording in vivo identified by electrolytic lesions stained by Nissl. (**d**), The variation of firing rate for identified dDpMe GABAergic neurons at state transitions.

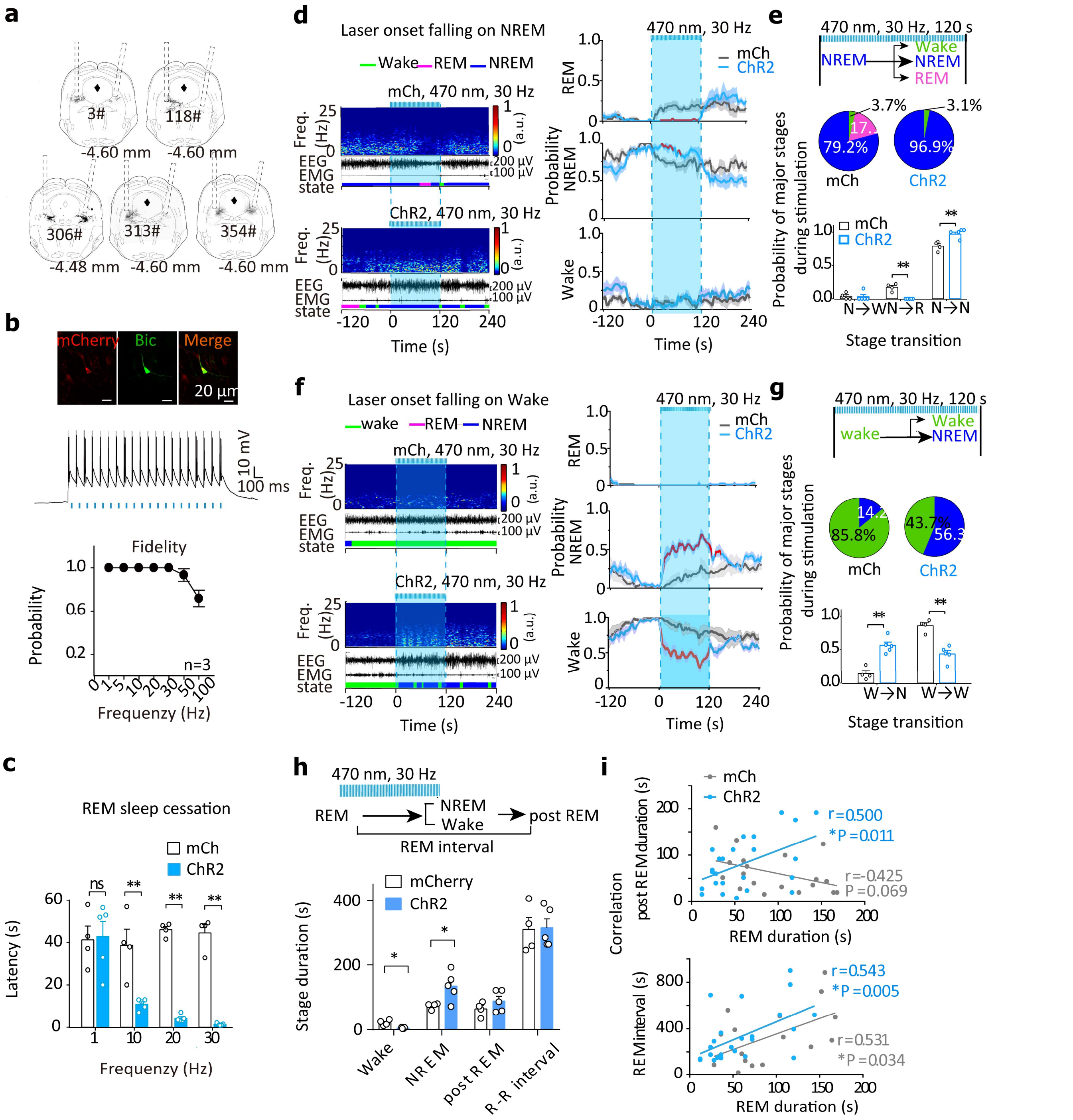

S2 Optogenetic activation of dDpMe GABAergic neurons suppresses REM sleep and promotes NREM sleep.

(a) Coronal sections showing the distribution of ChR2-mCherry-expressed neurons and optic tips in the dDpMe of each GAD2-IRES-Cre mice. (**b**) The typical electrophysiological characteristics and action potential trains induced by 470 nm laser pulse trains (5 ms per pulse) of the ChR2-mCherry cell in patch clamp. The fidelity of ChR2-mCherry expressing neuron tested by various frequencies of blue light photostimulation. (**c**) The latency of REM sleep cessation by different frequencies. Assessed by two-way ANOVA followed by Sidak’s multiple

comparisons test. $F_{1,28}$ = 79.06, P = 1.2 × 10^{-9}; 1 Hz, P = 1.0; 10 Hz, P = 5.0 × 10^{-4}; 20 Hz, P = 1.2 × 10^{-6}; 30 Hz, P =6.8 × 10^{-7}. (**d,f**) The probability of brain states in GAD2-IRES-Cre mice after bilateral laser onset falling on wakefulness (d) or NREM sleep (f) above the dDpMe. **(e**) The pie charts and histograms show changes in brain states during laser stimulation initiated from NREM sleep (NREM→REM, P = 2.3 × 10^{-12}). (**g**) The pie charts and histograms show changes in brain states during laser stimulation initiated from wakefulness (wake→NREM, P = 7.9 × 10^{-9}). (**h**) The change of subsequent stage mean duration after laser stimulation. *$P_{(wake)}$ = 4.0 × 10^{-3}; $P_{(NREM)}$ = 0.033. (**i**) The correlation analysis of suppressed REM sleep duration, post REM sleep duration, and REM-REM sleep interval in $dDpMe^{GABA}$ ChR2 group compared with control group. ChR2-mCherry group, n = 5; mCherry group, n = 4; *P < 0.05, **P < 0.01.

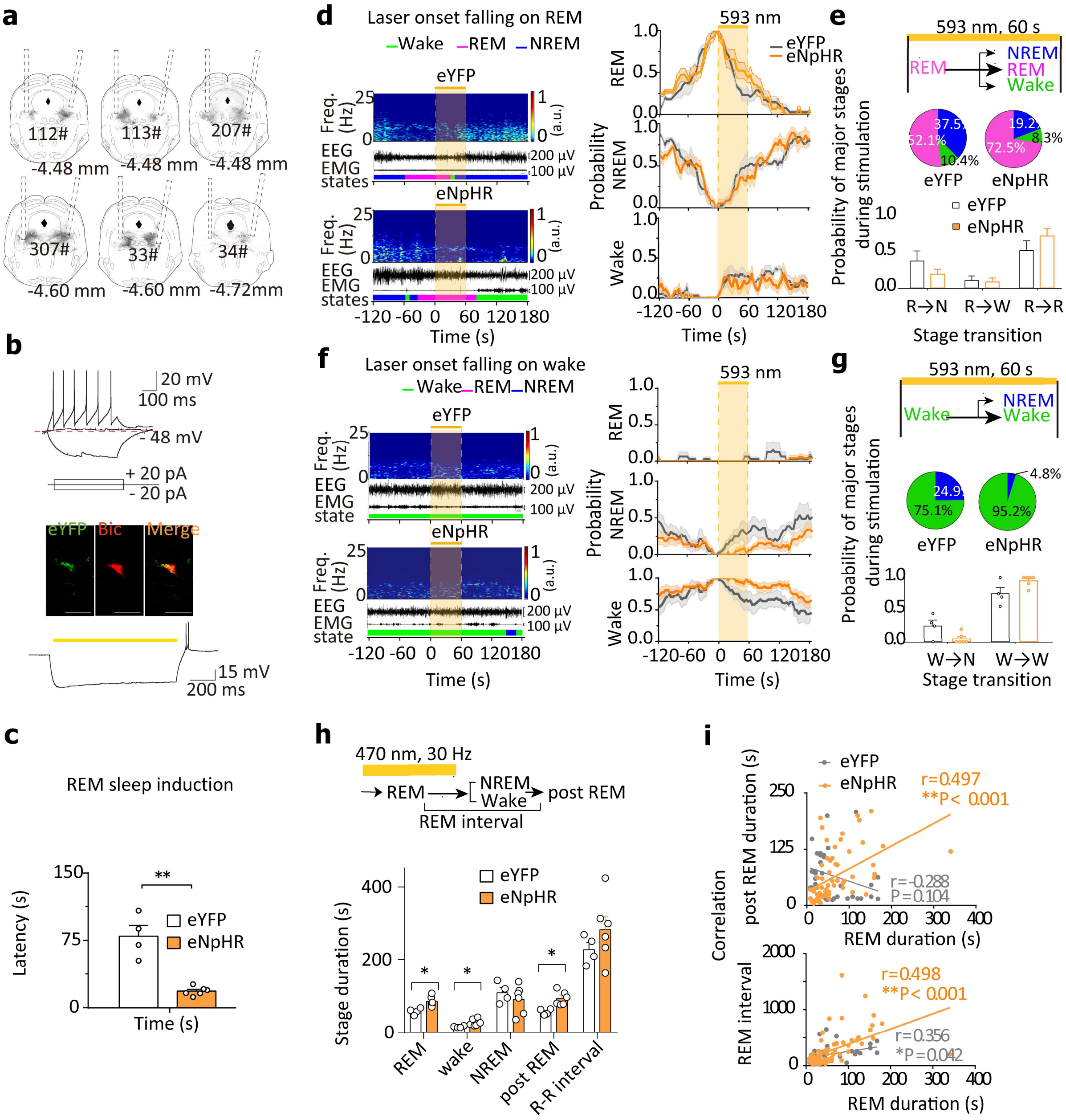

S3 Optogenetic inactivation of dDpMe GABAergic neurons induces excessive REM sleep and weakens NREM sleep.

(**a**) Coronal sections showing the distribution of eNpHR-eYFP -expressed neurons and optic tips in the dDpMe of each GAD2-IRES-Cre mice. (**b**) The typical electrophysiological characteristics of the eNpHR-eYFP cell. A persistent hyperpolarization of the typical eNpHR-eYFP cell (yellow) labelled by bioyctin (red) was induced by constant 593 nm laser exposure. (**c**), The latency of REM sleep induction and the change of subsequent stage mean duration after laser stimulation in eNpHR group compared with control group. Assessed by student's t test. *P*<0.001. (**d**,**f**) Example recording of EEG power spectrograms, EEG/EMG traces, hypnograms, and the probabilities of different brain states in GAD2-IRES-Cre mice after bilateral laser stimulation above the dDpMe

(593 nm, 60 s) in terms of REM sleep (d) or wake (f). (**e**,**g**) Pie charts and histograms showing major states during laser stimulation initiated from different brain states, which were assessed by two-tailed *t*-tests. (**h**) Changes of subsequent stage mean duration after laser stimulation, stage duration: Assessed by two-tailed student's t test, $P < 0.05$. $P_{(REM)} = 8.4 \times 10^{-3}$; $P_{(post\ REM)} = 0.016$. (**i**) The correlation of induced REM sleep duration, post REM sleep duration, and REM-REM sleep interval in dDpMe^{GABA} eNpHR group are measured by the Pearson product-moment correlation coefficient. eNpHR-eYFP group, n = 6; eYFP group, n = 4, *$P < 0.05$, **$P < 0.01$.

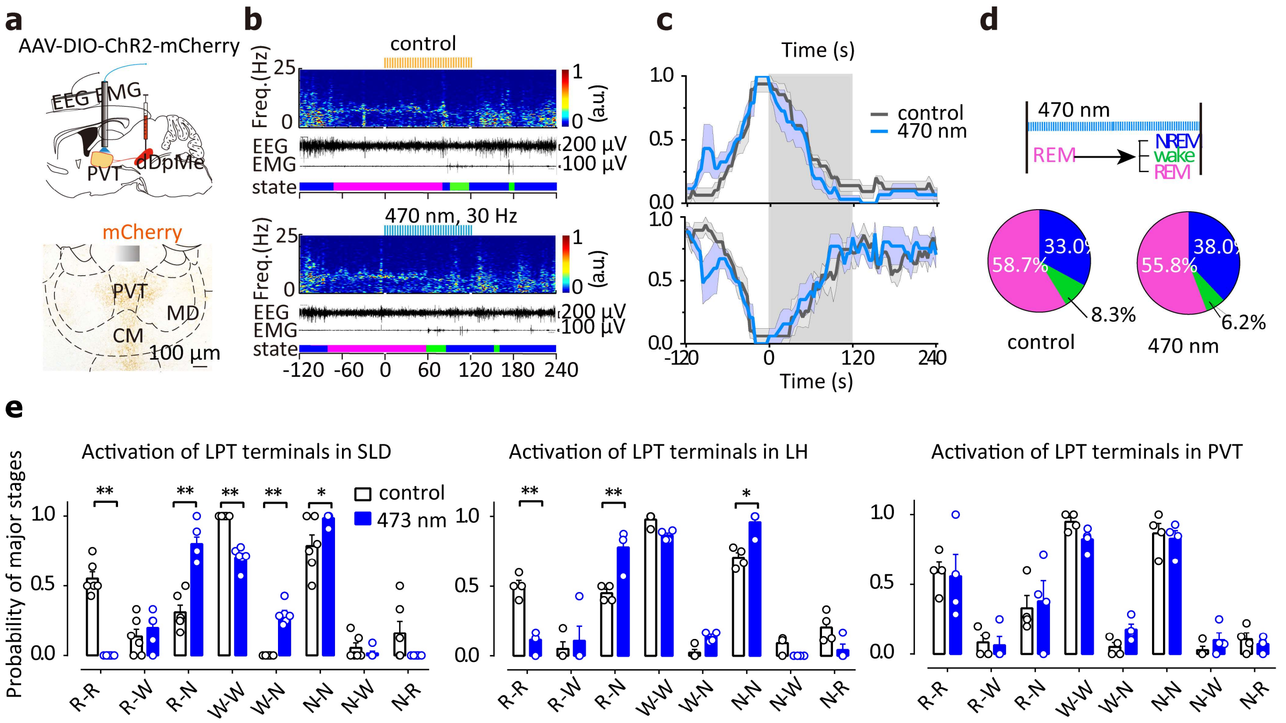

S4 Diverse change of brain states after activating different parts of dDpMe GABAergic neuronal axonal terminals.

(**a**) Schematic for optogenetic experiments and polygraphic recordings of the dDpMe-PVT pathway with dDpMe neurons expressing ChR2-mCherry in GAD2-IRES-Cre mice. (**b**) Example recordings of EEG power spectrograms, EEG/EMG traces, and hypnograms in GAD2-IRES-Cre mice after bilateral blue-light stimulation (bottom, 470 nm, 30 Hz, 5 ms, 120 s) or yellow-light stimulation (top, 593 nm, 30 Hz, 5 ms, 120 s) of dDpMe^{GABA} axonal terminals in the PVT. (**c**) Time courses of REM sleep (top) or NREM sleep (down) in GAD2-Cre mice after 593-nm or 470-nm laser stimulation of axonal terminals in the dDpMe or ChR2-mCherry expressing neurons in the PVT initiated from REM sleep. There's no significant difference assessed by two-way ANOVA (*post-hoc* Sidak's multiple-comparison tests) $F_{(1,\ 546)(REM)}$ = 1.31, P = 0.25; $F_{(1,\ 546)(NREM)}$ = 0.08, P = 0.77; $F_{(1,546)(wake)}$ = 2.33, P = 0.13. (**d**) Pie chart showing major states during laser stimulation initiated from REM sleep (ChR2-dDpMe-PVT group, n = 4). (**e**) The histograms showing the probability of major states initiating from different brain states during light delivery to the dDpMe^{GABA} terminals in SLD, LH, and PVT. Data represent mean ± SEM. *P < 0.05, **P < 0.01. Abbreviations: PVT, paraventricular thalamus nucleus; CM, central medial thalamic nucleus; MD, mediodorsal thalamic nucleus.

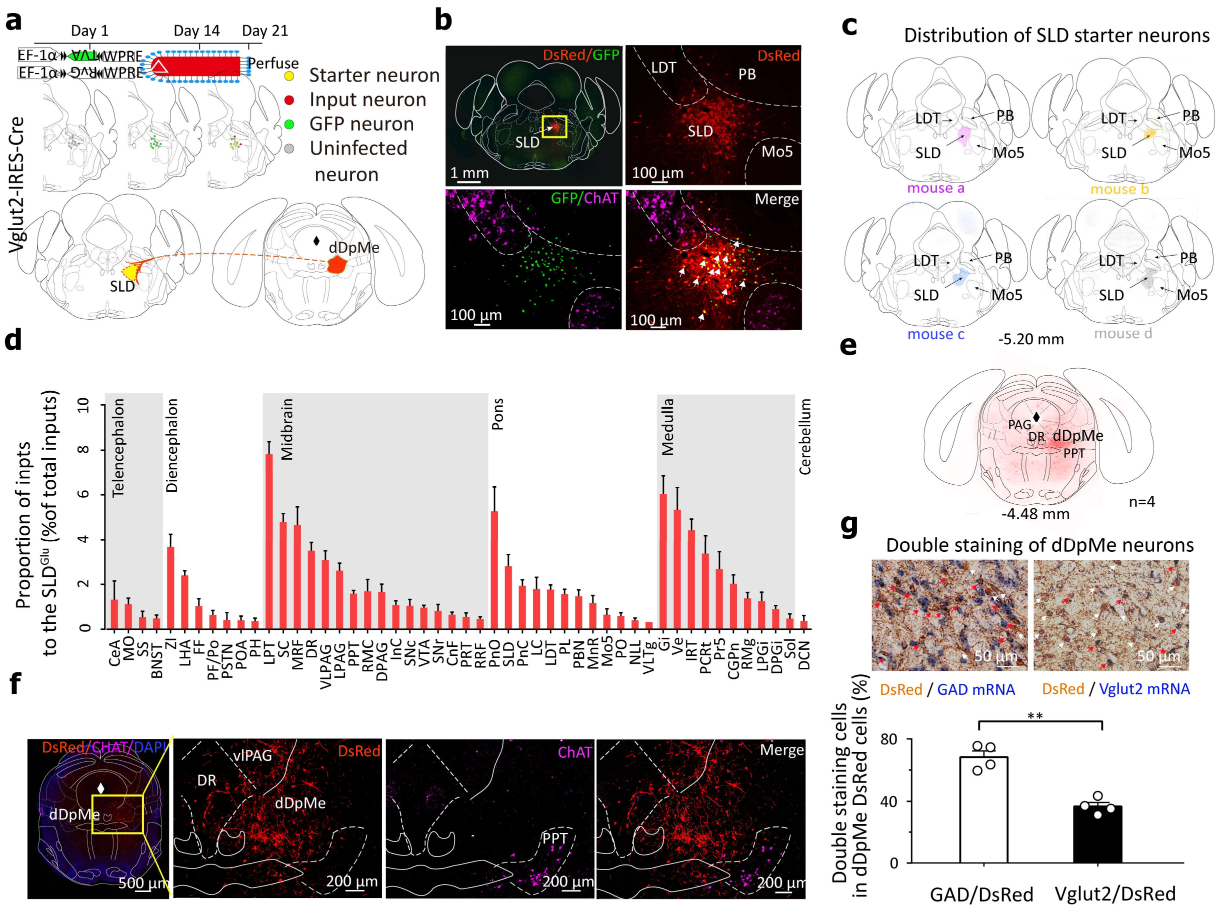

S5 dDpMe GABAergic neurons send the majority projections to SLD glutamatergic neurons. (**a**) An experimental schematic of the viral vectors and injection procedure for helper virus (AAV-EF1a-DIO-TVA-GFP and AAV-EF1α-DIO-RvG) and RV-EnvA-ΔRG-DsRed into the SLD of Vglut2-IRES-Cre mice. (**b**) Fluorescence images represented that viruses are restricted the unilateral SLD between the LDT and Mo5, which is labeled in violet by ChAT. And enlarged images of the yellow boxed region show that starter cells (marked by arrows) co-expressed GFP with DsRed. (**c**) The distribution of SLD starter neurons, which are represented by different color for each mouse. (**d**) Statistical analysis of whole-brain inputs to SLD glutamatergic neurons. Average proportion of cell number in each brain region with more than 0.2% average input proportions from SLD glutamatergic neurons. Error bars represent the s.e.m. (**e**) The superimposed images of four mice show numerous inputs neurons in the dDpMe. Double staining cells are marked by red arrows and simple staining DsRed cells are marked by white arrows. (**f**) An enlarged view of the yellow boxed region in the leftest image shows that a large number of the input neurons in the dDpMe. (**g**), Images of dDpMe co-labelled DsRed neurons with GAD1 or Vglut2 mRNA, and quantification of DsRed+ cells double staining for GABA and Glutamate in the dDpMe. n = 4, each data point represents one experimental animal. Significantly difference from dDpMe GAD1+ DsRed+/ DsRed+ neurons to Vglut2+DsRed+/ DsRed+ neurons assessed student's *t* test. Data represent mean ± SEM. **P* < 0.05, ***P* < 0.01. Abbreviations: LDT, laterodorsal tegmental nucleus; PB, parabrachial nucleus, Mo5, motor trigeminal nucleus; DR, dorsal raphe; PPT, pedunculopontine tegmental nucleus.

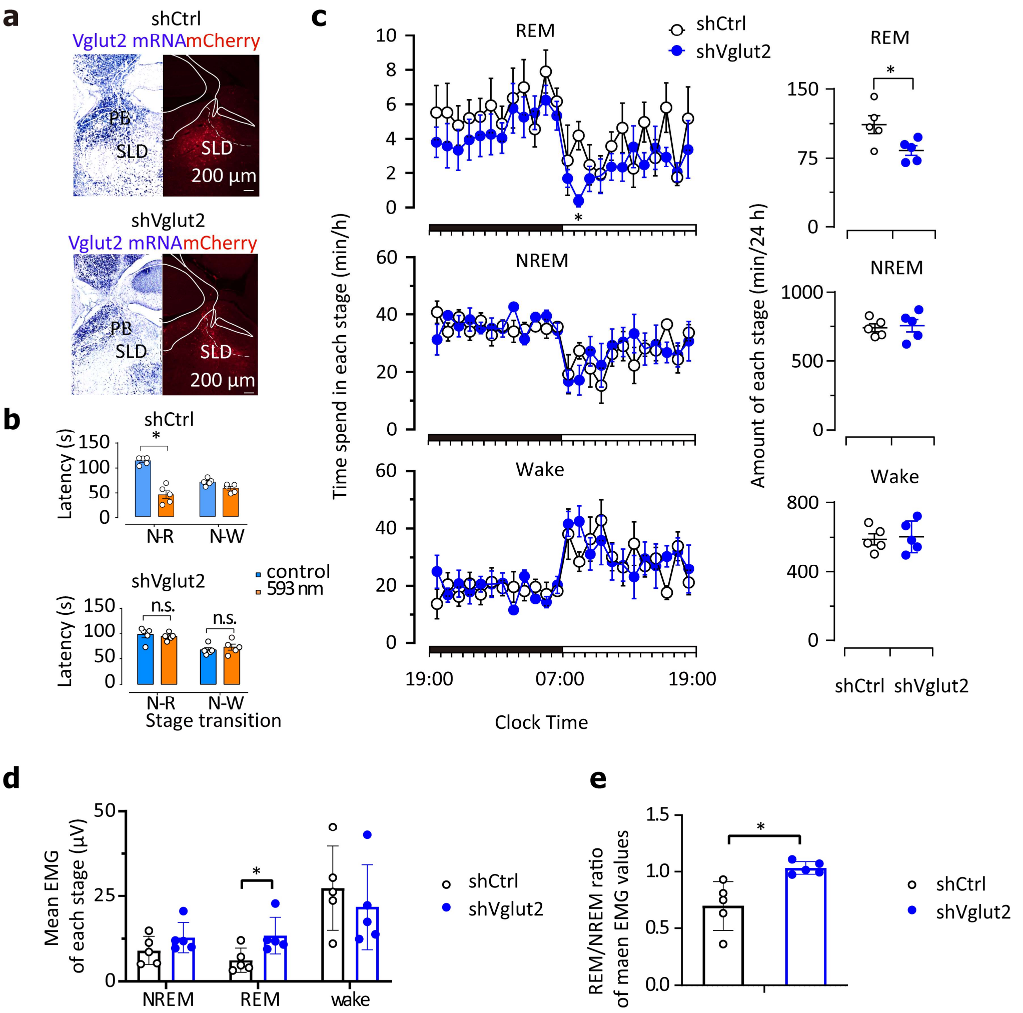

S6 Blocking glutamatergic neurotransmission in the SLD attenuated REM sleep and increased atonia.

(**a**) Fluorescent mCherry and *in-situ* hybridization to label Vglut2-mRNA to label shCtrl or shVglut2. (**b**) The latency of REM sleep or wakefulness induction by laser stimulation after shRNA interference by shCtrl ($F_{(1,16)}$ = 63.78, $P_{(NREM \rightarrow REM)} < 0.001$) or shVglut2 ($F_{(1,16)}$ = 0.02 $P_{(NREM \rightarrow REM)}$ = 0.83) was assessed by two way ANOVA with *post-hoc* Sidak's multiple-comparison tests. (**c**) Time course of brain states following SLD glutamate knock down by AAV-shVglut2 or injecting AAV-shCtrl. Significantly different from the corresponding control group assessed by two-way ANOVA (post-hoc: Sidak's multiple comparisons test. *: shVglut2 VS shCtrl, $F_{8,184}$ = 3.664 (NREM), 1.92 (REM), 2.34 (wake). Total amounts of brain states over 24-h administration. (Two-tailed *t* test. *: shVglut2 VS shCtrl, n = 5). (**d**) Mean EMG values during brain states in AAV-shCTRL (open circles) versus AAV- shVglut2 (filled circles) mice. (Two-tailed *t* test. $P < 0.05$). (e) REM sleep/NREM sleep ratio of mean EMG values in AAV-shCTRL versus AAV- shVglut2 mice. (Two-tailed *t* test. $P < 0.05$). Data represent mean ± SEM, *$P < 0.05$, **$P < 0.01$. n=5 each group. Abbreviations: LDT, laterodorsal tegmental nucleus; PB, parabrachial nucleus.

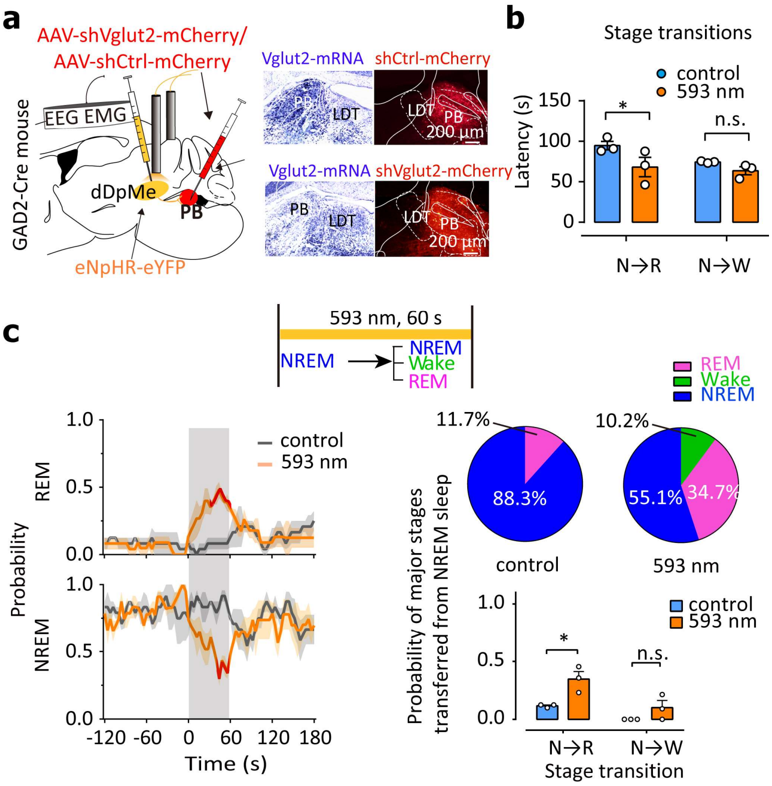

S7 dDpMe^{GABA}→PB^{Glu} neural pathway is not necessary for REM sleep termination.

(**a**) Sagittal diagram for optical inhibition of the dDpMe by eNpHR after shRNA interference of the PB by shVglut2. shRNA interference of Vglut2 in the PB is identified by *in situ* hybridization to label Vglut2-mRNA as well as fluorescent mCherry to label shCtrl (top) or shVglut2 (down). (**b**) The latency of REM sleep or wakefulness induction by laser stimulation after shRNA interference by shVglut2. (**c**) Time course of REM sleep (top) or NREM sleep (down) in GAD2-Cre mice with PB^{Glu} interference after 593 nm or 470 nm laser stimulation of dDpMe eNpHR-eYFP expressing neurons initiating from NREM sleep. The red lines indicate a statistical difference (*P* < 0.05, two-way ANOVA with post-hoc of the Sidak's multiple comparisons test). The pie chart and histograms show major states during laser stimulation initiating from NREM sleep in shVglut2 group. n = 3. Data represent mean ± SEM. **P* < 0.05, ***P* < 0.01. Abbreviations: LDT, laterodorsal tegmental nucleus; PB, parabrachial nucleus, Mo5, motor trigeminal nucleus.

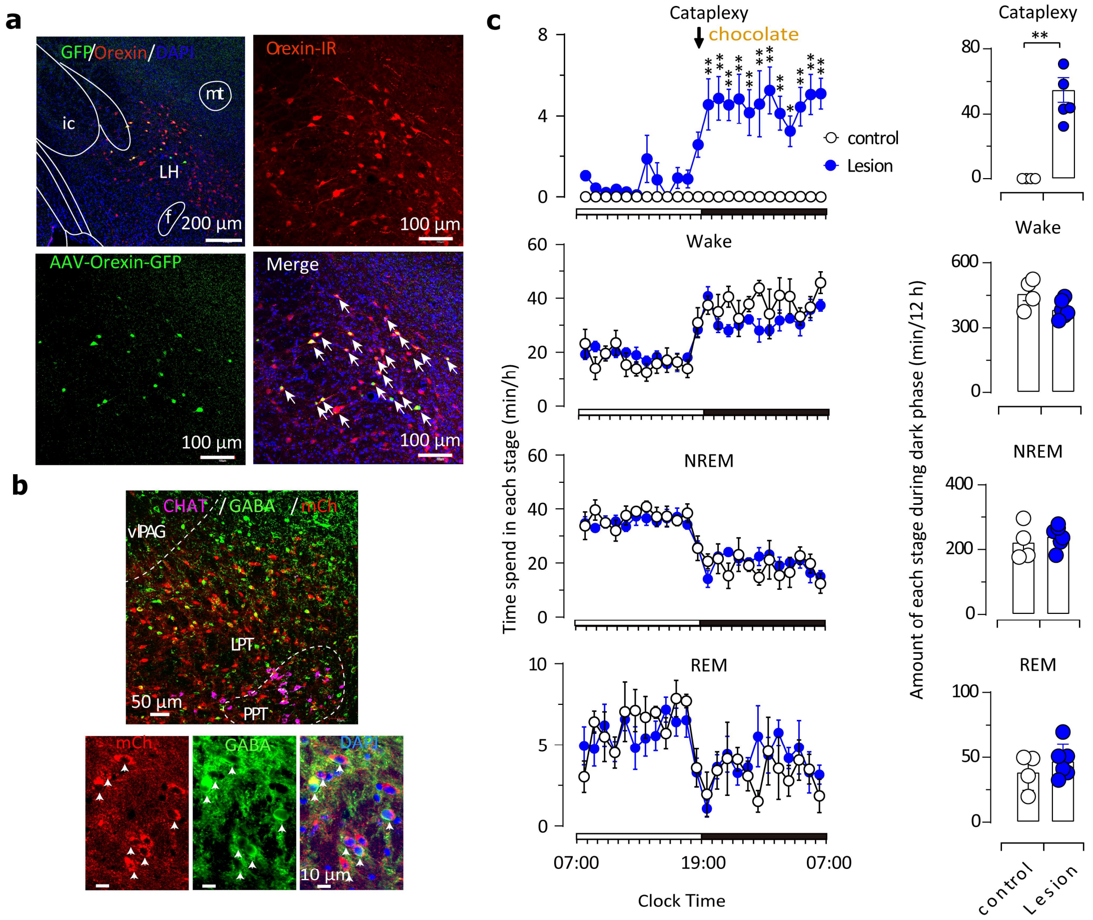

S8 Lesion of orexin neurons in the hypothalamus increased cataplexy and decreased wakefulness in dark phase.

(**a**) Fluorescence images represented that orexin neurons labeled in red are co-expressed with AAV-Orexin-Cre/AAV-EF1α-DIO-GFP viruses in green. And enlarged images show that the yellow neurons (marked by arrows) co-expressed GFP with Orexin antibody. (**b**) Typical fluorescent images representing the co-localization of dDpMe labeled by ChR2-mCherry, dorsomedial to the ChAT-labeled (purple) PPT, with GABA antibody expression (green). (**c**) Left, Time course of brain states following orexin neural lesion by caspase3. Significantly different from the corresponding control group assessed by two-way ANOVA (post-hoc: Sidak's multiple comparisons test. *: lesion VS control, $F_{1,192}$ = 152.8 (cataplexy), 13.31 (wake), 1.176 (NREM), 2.755 (REM). Right, Total amounts of brain states over 12-h administration during dark phase. (Two-tailed *t* test. *: lesion VS control, n = 4 ~ 6). Data represent mean ± SEM. *P < 0.05, **P < 0.01. Abbreviation: LH, lateral hypothalamus; ic, internal capsule; f, fornix.

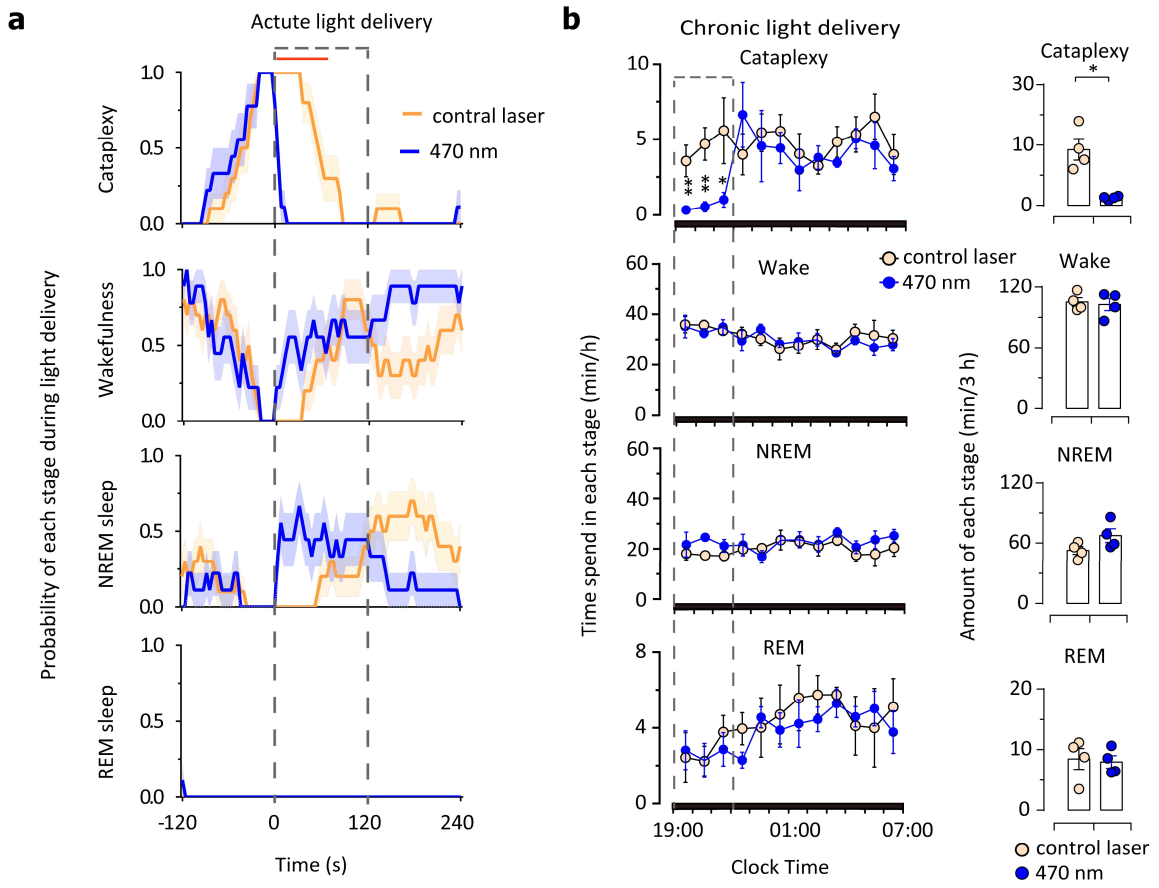

S9 Activation of dDpMe GABAergic neurons suppresses cataplexy.

(**a**) Time course of brain states in mice with orexin neural lesion after 593 nm (control) or 470 nm laser acute stimulation (30 Hz, 5 ms, 120 s) of dDpMe Vgat-ChR2-mCherry expressing neurons initiating from cataplexy attacks. The red lines indicates a statistical difference ($P < 0.05$, two-way ANOVA with post-hoc of the Sidak's multiple comparisons test). (**b**) Time course of brain states in mice with orexin neural lesion after 593 nm (control) or 470 nm laser chronic stimulation (30 Hz, 5 ms, 5 s per 30 s) of dDpMe Vgat-ChR2-mCherry expressing neurons for 3 hours in dark phase. Significantly different from the corresponding control group assessed by two-way ANOVA (post-hoc: Sidak's multiple comparisons test. *: lesion VS control, $F_{1,72}$ = 1.117 (cataplexy), 0.439 (wake), 4.78 (NREM), 0.928 (REM). Total amounts of brain states over 3-h administration during dark phase. (Two-tailed t test. *: lesion VS control, n = 4). Data represent mean ± SEM.

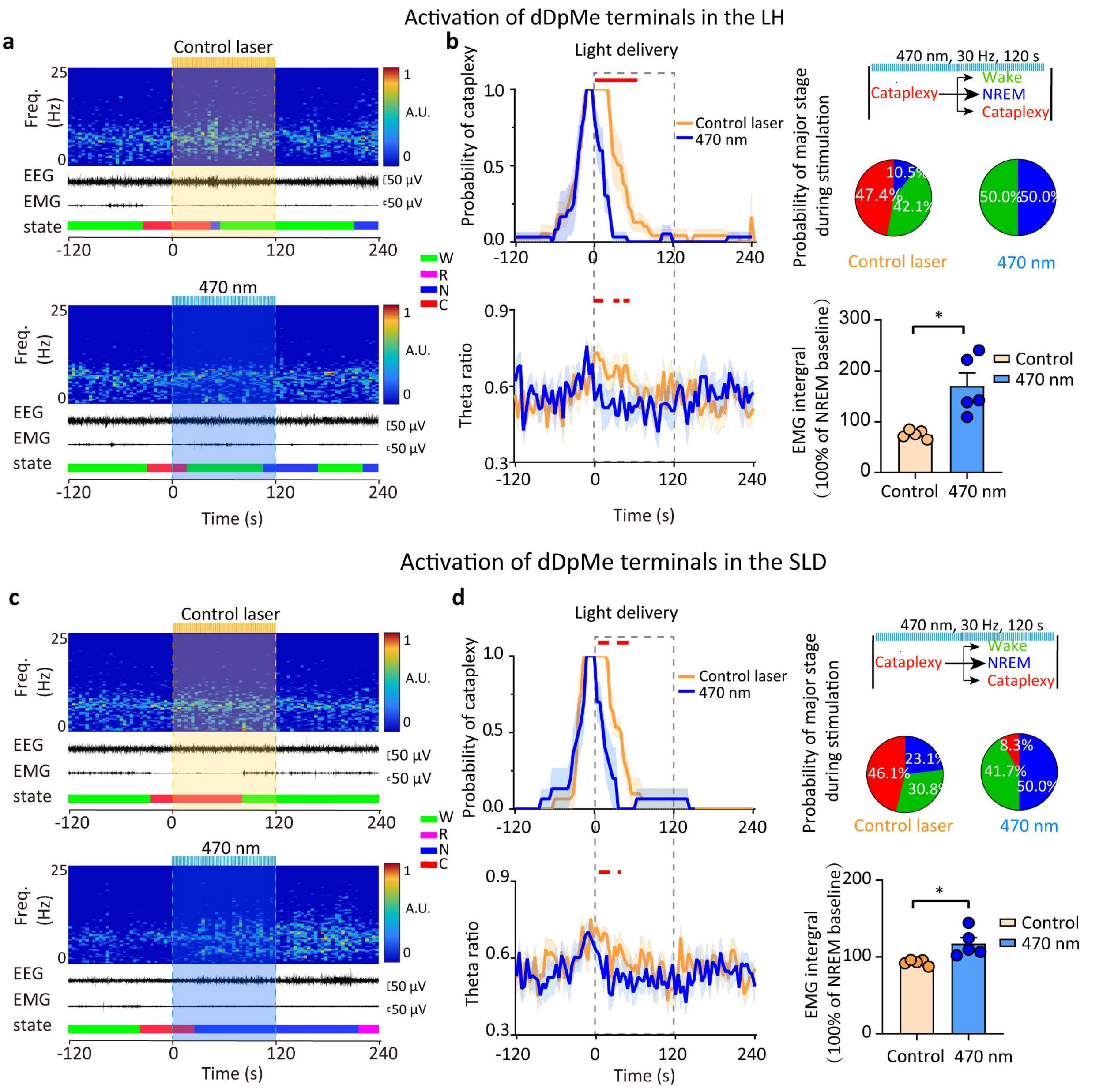

S10 Activation of dDpMe^{GABA} neuronal downstream suppresses cataplexy.

(**a,c**) Example recordings of EEG power spectrograms, EEG/EMG traces, and hypnograms in wild type mice, with bilateral blue-light stimulation (bottom, 470 nm, 30 Hz, 5 ms, 120 s) or yellow-light stimulation (top, 593 nm, 30 Hz, 5 ms, 120 s) of dDpMe^{GABA}-LH (a) or dDpMe^{GABA}-SLD (c). (**b,d**) Time courses of cataplexy, theta ratio, the probability of major stage, and EMG integral in wild type mice after 2 min control (593-nm) or 470-nm laser stimulation of dDpMe^{GABA}-LH (b) or dDpMe^{GABA}-SLD (d) terminals initiated from cataplexy. The red lines indicate a statistical difference between the 470 nm and control laser groups (*, $P < 0.05$). EMG integral assessed by Student's *t*-test (*, $P < 0.05$).

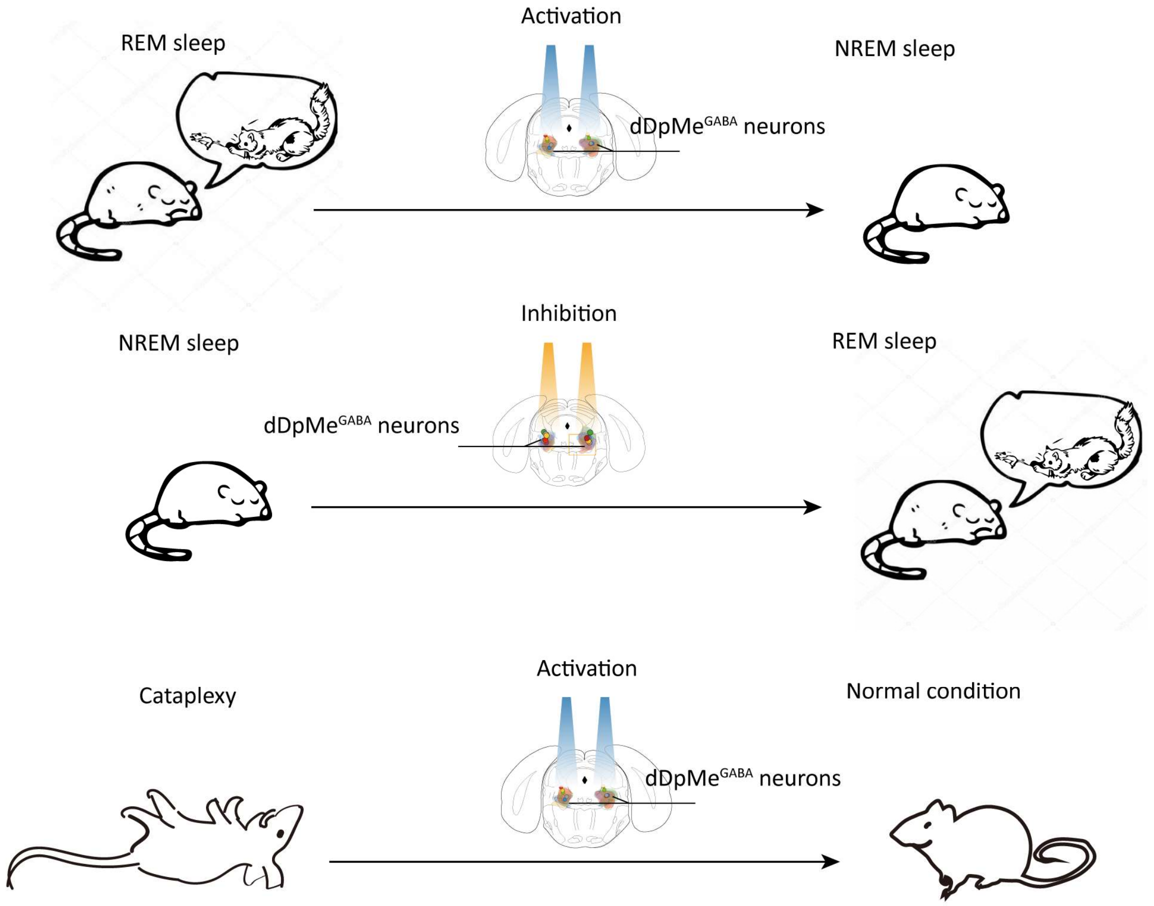

S11 The cartoon ideogram for vital roles of the dDpMe^{GABA} neurons in REM sleep and cataplexy suppression.

Top, activation of dDpMe^{GABA} neurons converts REM sleep or wakefulness to NREM sleep. Medium, inactivation of dDpMe^{GABA} neurons induces excessive REM sleep and promotes NREM-to-REM transitions. Bottom, activation of dDpMe^{GABA} neurons relieve cataplexy.

Movie S1. Cataplexy attacks occur in orexin-caspase-3 mice (106 s).

Movie S2. 470 nm-light delivery to dDpMe-Vgat-ChR2 neurons effectively and sharply curbed cataplexy attacks in orexin-caspase-3 mice (165 s).

Reference

1 Qu, W. M. *et al.* Essential role of dopamine D2 receptor in the maintenance of wakefulness, but not in homeostatic regulation of sleep, in mice. *J. Neurosci.* **30**, 4382-4389 (2010).

2 Yuan, X. S. *et al.* Striatal adenosine A2A receptor neurons control active-period sleep via parvalbumin neurons in external globus pallidus. *Elife* **6** (2017).

3 Chen, Z. K. *et al.* Whole-Brain Neural Connectivity to Lateral Pontine Tegmentum GABAergic Neurons in Mice. *Front. Neurosci.* **13**, 375 (2019).

4 Long, J. E., Cobos, I., Potter, G. B. & Rubenstein, J. L. Dlx1&2 and Mash1 transcription factors control MGE and CGE patterning and differentiation through parallel and overlapping pathways. *Cereb Cortex* **19 Suppl 1**, i96-106 (2009).

5 Yuan, M. Y. *et al.* Ablation of olfactory bulb glutamatergic neurons induces depressive-like behaviors and sleep disturbances in mice. *Psychopharmacology (Berl)* (2020).
